# Psychological adversities and epigenetic ageing in midlife and older age: A systematic review and meta-analysis

**DOI:** 10.1016/j.tjpad.2026.100650

**Published:** 2026-08-07

**Authors:** Jiuyu Guo, Jiatong Shan, Kaisy Xinhong Ye, Wei Liang, Yanyu Wang, Eng-Tat Ang, Tih-Shih Lee, John Suckling, Andrea B. Maier, Lei Feng

**Affiliations:** a Department of Psychological Medicine, Yong Loo Lin School of Medicine, National University of Singapore, Singapore; b Centre for Healthy Longevity, @AgeSingapore, National University Health System, Singapore, Singapore; c Healthy Longevity Translational Research Program, Yong Loo Lin School of Medicine, National University of Singapore, 119228, Singapore; d NUS Academy for Healthy Longevity, Yong Loo Lin School of Medicine, National University of Singapore, Singapore; e Kangning Hospital of Shenzhen, Luohu District, Shenzhen, China; f School of Psychology, Shandong Second Medical University, Weifang, China; g Alice Lee Center for Nursing Studies, Yong Loo Lin School of Medicine, National University of Singapore, Singapore; h Neuroscience and Behavioral Disorders Program, Duke-NUS Graduate Medical School, Singapore; i Brain Mapping Unit, Department of Psychiatry, University of Cambridge, Cambridge, UK

**Keywords:** Loneliness, Depression, Stress, Epigenetic age acceleration, DNA-m based age, Meta analysis

## Abstract

**Background:**

Epigenetic modification is a hallmark of aging that encloses physiological information relevant to health and longevity and has been used to construct epigenetic clocks that estimate epigenetic age acceleration (EAA) to monitor population health across the life span. Psychological adversities (PA) are recognized contributors to poor health; however, their potential role in EAA remains insufficiently understood in older adults.

**Objective:**

This study systematically reviews and meta-analyzes the association between PA and EAA from midlife onward.

**Methods:**

Eligible literatures were indexed in five databases up to July 2025. Study quality was assessed using an adapted Newcastle-Ottawa scale. Meta-analyses were performed using random-effects models, followed by post hoc and sensitivity analyses to assess robustness.

**Results:**

Twenty-two studies were included, of which fifteen were classified as high quality. Irrespective of the type of psychological adversity, positive associations were consistently observed for second-generation epigenetic clocks (PhenoAge and GrimAge). Meta-analyses revealed that greater loneliness (β = 0.07, 95 % CI [0.06, 0.08], *I*^2^ = 0 %, p = 0.002), depression (β = 0.08, 95 % CI [0.04, 0.13], *I*^2^ = 55.2 %, p = 0.003), and stress (β = 0.10, 95 % CI [0.03, 0.16], I2 = 68.4 %, p = 0.009) were each associated with higher EAA.

**Conclusions:**

Psychosocial stress, depression, and loneliness are each associated with accelerated aging from midlife onward. Notable gaps include the lack of studies examining anxiety and underrepresentation of non-Western population. Whether alleviating psychological adversities translates into decelerated aging trajectories requests future intervention studies.

## Highlights

• Each SD rise in loneliness associates with 0.07 SD higher EAA in midlife +.
• Each SD rise in depression associates with 0.08 SD higher EAA in midlife +.
• Each SD rise in stress associates with 0.10 SD higher EAA in midlife +.
• The stress-EAA association seems to be stronger in midlife relative to older adults.

## 1 Introduction

The aging population [1,2] presents noticeable societal challenges worldwide. Advances in healthcare and technology have drastically extended life expectancy in recent decades. However, longer life expectancy does not necessarily translate into a better quality of life in older age. Aging is an intricate physiological process [3] and remains a primary risk factor for various chronic conditions, diseases, as well as poor health [4]. Promoting healthy aging (HA) is therefore essential to mitigate age-associated declines and preserve independence [5,6], thereby enabling older adults to continuously function in later years.

Psychological well-being is a consistent indicator of HA [7]. Although conceptually distinct, loneliness, anxiety, depression, and stress (LADS) represent interrelated components of psychological adversities (PA) that may influence aging via both overlapped and domain-specific pathways. Globally, approximately one in every four older adults currently experiences LADS [[8], [9], [10]]. Although these adversities are established risk factors for adverse outcomes such as cognitive decline, cardiovascular diseases, frailty, compromised immunity, and all-cause mortality [[11], [12], [13], [14], [15], [16], [17]], their potential extent to which they may embed in biological aging remains insufficiently studied. In this review, psychological adversity (PA) is employed as an umbrella term of LADS phenotypes, without implying a unitary construct.

### 1.1 Epigenetic aging

Epigenetic modification is one primary hallmark of biological aging [3]. Epigenetic regulation of DNA transcription, gene expression, and downstream function involves direct modifications and coordination of DNA, without altering the genetic sequence. The most widely studied mechanism is DNA methylation, particularly an additional methyl group on the fifth carbon of cytosine (5-methylcytosine). Prior aging literature have linked chronological age to de novo methylation at Cytosine-phosphate-Guanine dinucleotide islands (CpGIs, CG-rich regions often found near promoter regions), but with a global loss of methylation patterns across individual Cytosine-phosphate-Guanine dinucleotides (CpGs). These age-related patterns enclose important physiological information and have directly informed the development of *epigenetic clocks*. Briefly, the first-generation (e.g., HorvathAge, HannumAge) were each trained to predict chronological age [18,19], while the second-generation (e.g., PhenoAge, GrimAge) incorporated additional biomarkers to forecast health span [20,21]. The third generation (e.g., DunedinPACE) quantifies the aging rate relative to calendar year [22]. Epigenetic age acceleration (EAA), defined as the residual from regressing DNA methylation-based age on chronological age, is commonly utilized to indicate whether an individual appears epigenetically younger or older than expected for their chronological age.

Psychological symptomatology and its underlying mechanisms indeed vary substantially across the life span [[23], [24], [25], [26], [27]]. Midlife, commonly defined as beginning around 40 years of age, represents a crossroad marked by heightened vulnerability to psychological distress and accelerated aging trajectory [28,29]. Additionally, greater symptom severity and suicidal thoughts are observed in older adulthood compared to younger counterparts [24,26]. These patterns suggest that PA and biological aging may interact in a mutually reinforcing manner, potentially amplifying adverse outcomes in older age.

Given these age-related variations, the present study focuses specifically on midlife and older adults. We systematically reviewed and meta-analyzed the eligible literature in adherence to the PRISMA protocol [30]. The primary objectives were to: (i). examine associations between loneliness, anxiety, depression, stress, and epigenetic aging from midlife onwards, and (b) explore variations in these associations across subgroups such as clinical versus subsyndromal samples and midlife versus older adults.

## 2 Methods

This study was prospectively registered with the International Prospective Register of Systematic Reviews (PROSPERO; ID: CRD42024572630).

### 2.1 Database search

The search strategy is detailed in **Appendix Table 1**. An electronic database search was conducted in five databases (e.g., PubMed, Scopus, Embase, Web of Science, and PsycINFO (via OVID)). A comprehensive list of keywords was developed and combined for advanced searches, with filters applied as applicable. The most recent search was indexed up to 20th June 2025.

### 2.2 Inclusion criteria

Eligible studies were those analyzing associations between loneliness, anxiety, depression, or stress and epigenetic aging. Any DNA methylation-based epigenetic clock was eligible, irrespective of the biological tissue from which the DNA was extracted. For customized clocks, a description of the selected cytosine-phosphate-guanine dinucleotides (CpGs) and the statistical approach should be provided to facilitate transparency. Additionally, a description of items and psychometric properties was needed if an adapted measurement was employed. Studies with a mean chronological age of 40 years and above were deemed eligible, reflecting a study-level midlife cutoff rather than a strict individual cutoff [31,32]. Age range and the proportion of participants under 40 were extracted when reported, with the potential impact of age-range variation examined in sensitivity analyses.

There were no restrictions regarding study type, design, or the clinical status of anxiety or depression. Studies were included if participants had a diagnosed depressive disorder and/or anxiety disorder, either through explicit enrollment or if diagnoses were made by trained psychiatrists based on standardized criteria (e.g., DSM, ICD).

### 2.3 Exclusion criteria

Studies reporting only the reverse-direction association, in which EAA predisposed subsequent psychological outcomes, were excluded because the preregistered PROEPSECO defined PA as the primary exposure and EAA as the outcome. If any, studies were retained in the table of study exclusion and narratively discussed. We also excluded studies if participants had concurrent psychotic disorders or if psychological adversities arose from terminal illnesses. Non-English language publications, conference papers, review articles, and in vitro studies were also excluded.

### 2.4 Study selection

Records were imported into EndNote 20 for duplicate removal. The eligibility assessment was subsequently conducted using Rayyan, where two reviewers (JG, JS) independently screened the titles and abstracts to exclude irrelevant records. The full-text screening was independently conducted by the same reviewers (JG, JS) afterward. Any conflicts were resolved through discussions, and rationales for excluded studies were recorded and reported in the reasons for exclusion table (**Appendix Table 2**).

### 2.5 Data extraction

A standardized data sheet was developed to extract the relevant data from the included studies. Two reviewers (JG, WL) independently extracted data, manually from graphical information using WebPlotDigitizer, where appropriate. Discrepancies were resolved through discussion and, where necessary, with a third reviewer (LF).

Results from the source literature on the psychological adversities of interest were extracted, including instrument tools, cutoff scores (if any), and diagnosed conditions. We distinguished the clinical/registry cohort from the elevated/subsyndromal group. When participants were categorized into groups based on a prespecified cutoff, the number in each group was recorded. For outcomes, we collected the mean and SD of the estimated epigenetic age (EA) and epigenetic age acceleration (EAA) when available.

Statistics from the regression models were collated. These included beta coefficients (unstandardized *b* or standardized *β*), adjusted covariates, standard errors (SE), confidence intervals (95 % CI), and statistical power (i.e., *p*-values). To ensure consistency and to minimize redundancy, data were extracted from both unadjusted models and an additional adjusted model with common covariates. This enhanced comparability across studies while also mitigating the risk of overfitting.

Study characteristics were recorded, including study identification (author, year of publication), cohort name, country, mean chronological age of participants (mean, SD), size of the analytical sample, sex distribution (*n* [%], biological tissue or DNA source, epigenetic clocks, and analytical design.

### 2.6 Quality assessment

The Newcastle-Ottawa Scale (NOS) was adapted to evaluate study quality across three domains: selection, comparability, and outcomes, with a maximum of nine stars. Selection evaluated cohort/source description, exposure assessment, and biological sample (i.e., methylation-method) reporting. The comparability section evaluated adjustment for covariates to facilitate across-studies comparability. Lastly, the outcome section considered the appropriateness and transparency of statistical analyses and the adequacy of the study period. Studies were categorized as high quality (≥ 7 stars), moderate quality (≥ 5 stars), and low quality (< 5 stars), consistent with conventional NOS thresholds. Noting these ratings reflect reporting and association-study quality, they should not be interpreted as evidence of causal validity. Two reviewers (JG, KXY) independently administered the assessment, and discrepancies were resolved by a third reviewer (LF).

### 2.7 Meta-analysis

All statistical analyses were performed in RStudio (version 4.5.1) under a predetermined seed. Effect sizes were fully standardized to ensure comparability across studies. Standardization utilized the reported SD of psychological measures and EAA. When SD_EAA_ was not explicitly reported, but the SD_chronological age_ and SD_epigenetic age_ were available, SD_EAA_ was approximated using the following formula. When SD_epigenetic age_ was not provided, *r* = 0.82 was selected *a priori* for SD_EAA_ approximation based on large validation datasets from the original GrimAge development study by Lu et al. [21]. When regression was not performed, effect sizes were obtained based on (a) group sample sizes for prespecified categorical comparisons, and (b) reported means (μ) and SD_EAA_. Standard errors were computed using confidence intervals if not explicitly provided.

$$\sigma_{Epigeneticageacceleration}\;=\sqrt{\;\sigma_{chronological\;age}^{2}+\;\sigma_{epigenetic\;age}^{2}-\;2\;\times \;cov_{\left(chronological\;age,\;epigenetic\;age\right)}}$$

where $cov_{\;(chronological\;age,\;epigenetic\;age)}$ = $\sigma_{chronological\;age}$ × $\sigma_{epigenetic\;age}$ × *r*

To preserve statistical independence, only one effect size per study was included in the primary meta-analyses. The clock hierarchy was specified *a priori* to prioritize clocks with stronger links to health span and mortality [33,34]. GrimAge was selected when available, followed by PhenoAge. If neither was available, the most comparable DNA methylation-based age acceleration metric (e.g., Intrinsic epigenetic age acceleration (IEAA), extrinsic epigenetic age acceleration (EEAA)) was used. Customized clocks were used only when standard clocks were unavailable and sufficient methodological details were reported. Because within-person changes of different clock estimates were unavailable for most studies and the number of studies per domain was small, multilevel models with clock effects nested within studies were not performed. Instead, clock-specific meta-analyses and a study-level direction/significance table were used as sensitivity and descriptive analyses. Among longitudinal studies reporting multiple effects across waves, composite effect sizes and variances were approximated using the formula [35].

Random-effects models were used to pool the group effect sizes using an inverse-variance method. The variance of the true effect size, *τ*^2^, was estimated using a restricted maximum likelihood estimator, and the Hartung-Knapp method was applied to compute pooled confidence intervals (CIs) around group effect sizes. These approaches have been cited as conservative in mitigating type I errors [36]. Heterogeneity was assessed using multiple indices, including weighted sum of squares (i.e., Cochrane Q), the *I*^2^ statistic, and the *τ*^2^ statistic. Where substantial heterogeneity was observed (*I*^2^ > 50 %), post hoc analyses, including influential case analyses, and an exploratory-based analysis, Graphic Display of Study Heterogeneity (GOSH), were conducted to examine the robustness and stability of pooled effect sizes across all possible study subsets (2^k−1^ combinations). To further investigate sources of heterogeneity, meta-regressions were performed using mixed-effect models. Subgroups were coded as dummy variables in the regression models. Model fit was assessed using the proportion of residual heterogeneity and the variance explained by moderators (R^2^*).

To assess robustness, a comprehensive set of sensitivity analyses was conducted. These included (i) clock-specific meta-analyses, (ii) refitting models after omitting potential outliers or whose standardized effects were derived using approximated standard deviations, and (iii) sensitivity analyses addressing small-study effects.

#### 2.7.1 Publication bias

Publication bias was assessed using the small-study effect method and *p*-curve analysis exploratorily due to the limited number of studies. For methodological details of *p-*curve analysis, see [37]. Contour-enhanced funnel plots with shaded regions indicating different levels of significance were generated to visually inspect asymmetry. Egger’s test and trim-and-fill analyses were to adjust for potential funnel plot asymmetry. *P*-curve analysis was performed only when at least *K* = 5 statistically significant results (*p* < 0.05) were available for a respective meta-analysis, as a suggested minimum study number [37]. Given the small K, p-curve results were treated as exploratory and are reported in the Supplementary Material.

## 3 Results

### 3.1 Study characteristics

**Fig. 1:**
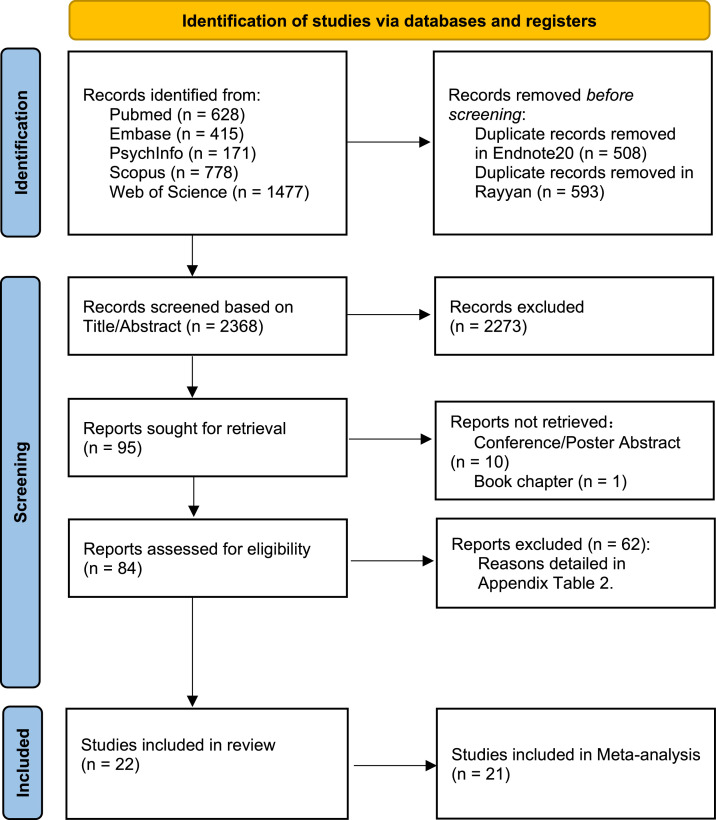
Study selection.

The study selection is shown in Fig. 1. The search returned 3469 records across five databases, of which 1101 were duplicates. The first screening based on titles and abstracts excluded 2273 irrelevant records. and 14 records that were abstracts or book chapters. The independent full-text assessment was conducted based on 84 studies, of which 62 were excluded (Appendix Table 2). A total of 22 studies were included and reported, of which 21 were eligible for meta-analysis. The exclusion was due to only a sex-stratified significant effect being reported, without an adequate study-level estimate [38]. The characteristics of the included studies are summarized in Table 1, and the literature findings are summarized in Table 2. A table summarizing of study-level classifications of significant and non-significant associations between PA and epigenetic clocks is available (**Appendix Table 3)**.

**Table 1:** Characteristics of included studies.

Author (Year)	**Cohort (Country)**	**N**	**Age**	**Male %**	**Domain**	**Exposure measure, reference period, cutoff**	**Tissue / DNA source**	**Clocks included**	**Design**
Bergquist et al. (2022)	United States	47	53.9	87.2 %	Stress	Perceived Stress Scale; recent perceived stress; high stress ≥ 12	Peripheral blood DNAm (as reported)	GrimAge	Cross-sectional
Boen et al. (2023)	Health and Retirement Study (United States)	6782	70.91	41.8 %	Stress	Cumulative stress burden; life-course/retrospective burden	Peripheral blood DNAm	PhenoAge; Klemera-Doubal method biological age	Cross-sectional
Freni-Sterrantino et al. (2022)	Northern Finland Birth Cohort (Finland)	604	46	44.2 %	Stress	Job strain; work-related stress	Peripheral blood DNAm	HorvathAge; HannumAge; PhenoAge; GrimAge	Cross-sectional
Markon et al. (2024)	Midlife in the United States (United States)	1310	Core 55.15; refresher 52.89	40.0 %	Stress	Psychosocial stress; longitudinal psychosocioeconomic stress/status	Peripheral blood DNAm	HorvathAge; HannumAge; GrimAge; PhenoAge; DunedinPACE	Longitudinal
Nwanaji-Enwerem et al. (2023)	VA Normative Aging Study (United States)	607	73.2	100 %	Stress	Perceived Stress Scale; recent perceived stress	Peripheral blood DNAm	HorvathAge; HannumAge; PhenoAge; GrimAge; SkinBlood clock; IEAA; EEAA	Longitudinal
Skinner et al. (2023)	Women's Health Initiative (United States)	3701	64.49	0 %	Stress	Stressful life events questionnaire; low 0–2, high 3–33	Peripheral blood DNAm	PhenoAge; GrimAge; IEAA; EEAA	Cross-sectional
Suglia et al. (2024)	Development Studies Disparities Study-Aging (United States)	359	49.35	53.2 %	Stress	Stressor Life Period Composite Score; childhood/adulthood/life-course	Peripheral blood DNAm	HorvathAge; HannumAge; GrimAge; PhenoAge; DunedinPACE; SkinBloodAge	Cross-sectional
Vetter et al. (2022)	BASE-II (Germany)	1100	75.60	47.91 %	Stress	Perceived Stress Scale; recent perceived stress	Peripheral blood DNAm	7CpGs; HorvathAge; HannumAge; PhenoAge; GrimAge	Cross-sectional
Zannas et al. (2015)	Grady Trauma Project (United States)	393	41.33	29.3 %	Stress	Stressful life events questionnaire; lifetime stressors	Peripheral blood DNAm	HorvathAge	Cross-sectional
Brown et al. (2017)	Health ABC Study (United States)	2776	73.58	48.8 %	Depression	CES-D; past-week depressive symptoms; significant symptoms > 10	Peripheral blood DNAm	LevineAge	Cross-sectional
Han et al. (2018)	Netherlands Study of Depression and Anxiety (Netherlands)	1130	41.5	35.5 %	Depression	IDS-SR 30-item; MDD/elevated symptoms ≥ 14; post-mortem replication narrative	Peripheral blood DNAm; post-mortem brain tissue analyzed narratively	Custom MBD-Seq-based clock	Cross-sectional
Joshi et al. (2024)	Canadian Longitudinal Study on Aging (Canada)	1479	59.7	49.5 %	Depression	CES-D 10-item; past-week depressive symptoms	Peripheral blood DNAm	HorvathAge; HannumAge; PhenoAge; GrimAge	Cross-sectional
Liu et al. (2022)	Emory Twin Study (United States)	467	Baseline 56; follow-up 67.6	100 %	Depression	BDI-II; past two-week depressive symptoms	Peripheral blood DNAm	HorvathAge; HannumAge; PhenoAge; GrimAge; IEAA; EEAA	Longitudinal (2 waves; mean follow-up 11.7 years)
Protsenko et al. (2021)	United States	109	40	42.2 %	Depression	IDS-SR and Hamilton Depression Rating Scale within MDD; diagnosed MDD case-control	Peripheral blood DNAm	GrimAge	Cross-sectional
Roberts et al. (2024)	Nurses' Health Study (United States)	760	44.2	0 %	Depression	Five-item Mental Health Index and CES-D; probable depression MHI-5 ≤ 60 or CES-D ≥ 10	Peripheral blood DNAm	HorvathAge; HannumAge; PhenoAge; GrimAge; DunedinPACE; DunedinPoAm	Longitudinal
Simons et al. (2023)	FACHS (United States)	693	58.1	58.1 %	Depression; Loneliness	Depression: mini-MASQ; Loneliness: UCLA Loneliness Scale	Blood-derived DNAm cortical-age proxy	Cortical BrainAge	Longitudinal
Sorli et al. (2024)	PREDIMED Plus-Valencia Study (Spain)	414	65.2	42.58 %	Depression	BDI-II; minimal 0–13, mild 14–19, moderate 20–28, severe 29–30	Peripheral blood DNAm	DunedinPACE	Cross-sectional
Tanifuji et al. (2023)	Gene Expression Omnibus database	699	>40 in study description	26.40 %	Depression	MDD case-control status from dataset metadata; scale/cutoff NR in Table 1 source	Blood DNAm dataset (GEO; tissue as reported in source)	HorvathAge; HannumAge; PhenoAge; GrimAge; SkinBloodAge	Longitudinal (9-year follow-up)
Wang et al. (2024)	Health and Retirement Study (United States)	3382	69.8	42.0 %	Depression	CES-D 10-item; low symptoms < 4, elevated/probable symptoms ≥ 4	Peripheral blood DNAm	HorvathAge; HannumAge; PhenoAge; GrimAge; DunedinPoAm	Cross-sectional
Beach et al. (2022)	FACHS (United States)	685	Baseline 48.77; follow-up 57.14	NR	Loneliness	UCLA Loneliness Scale; longitudinal loneliness	Peripheral blood DNAm	DunedinPACE	Longitudinal
Freilich et al. (2024) - MIDUS	Midlife in the United States (United States)	1310	54	44.60 %	Loneliness	Three-item loneliness scale; recent subjective loneliness	Peripheral blood DNAm	HorvathAge; HannumAge; PhenoAge; GrimAge; DunedinPACE; EAA average	Cross-sectional
Freilich et al. (2024) - HRS	Health and Retirement Study (United States)	NR	63.7–72.3 across 2010–2020 waves	41.50 %	Loneliness	UCLA Loneliness Scale; longitudinal loneliness	Peripheral blood DNAm	HorvathAge; HannumAge; PhenoAge; GrimAge; DunedinPACE; EAA average	Longitudinal (3 waves)

Note: NR = not explicitly reported; DNAm = DNA methylation; IEAA = intrinsic epigenetic age acceleration; EEAA = extrinsic epigenetic age acceleration.

**Table 2:** Summary of literature findings.

Study ID (Author, Year)	**Exposure of interest**			**Epigenetic Clock Analyzed**	**Estimated Epigenetic Age (mean, SD/median, IQR)**	**Model Estimation**			**Main Findings**	**Remarks**
	**Category**	**Measurement**	**Scale Outcomes (mean, SD/based on cutoff)**			**Unadjusted**	**Adjusted**	**Covariates**		
Boen et al. (2023)	Stress	PSS	0.128	PhenoAge, Klemera-Doubal method biological age	PhenoAge:0.635^a^ KDM bio-age:1.508^a^		PhenoEAA: *b* = 0.705 (SE = 0.125, *p* < 0.001) KDMEAA: *b* = 1.972 (SE = 0.362, *p* < 0.001)	Age, sex, birth cohort, region of birth, black * age female * age	Stress burden is positively associated with advanced PhenoAge acceleration at all stress levels. Regardless of stress levels, individuals of black race exhibit higher PhenoEAA.	
Bergquist et al. (2022)	Stress	Cumulative stress burden	11.7(5.4)	GrimAge	54.8(7.3)^b^	GrimAgeΔ: *b* = 0.239(*p* = 0.023, stress variable continuous)	GrimAgeΔ: Low Stress & Low Resilience: *b* = 3.185(95 % CI [0.084,6.286], *p* = 0.044)	Age, cell proportions	The reference group consists of participants with low stress and high resilience. A direct association between stress (measured as continuous) and GrimAgeΔ was revealed in an unadjusted model. Low stress and Low resilience were significantly associated with increased GrimAgeΔ compared to the reference. This association was reduced by half in those with high stress, low resilience, where it was no longer significant. This counterintuition suggests potential protective effects of certain stress levels in prompting survival and decelerating aging.	**∅**
Freni-Sterrantio et al. (2022)	Stress	Job Strain	0.19(0.44)	HorvathAge, HannumAge, PhenoAge, GrimAge	HorvathAge: 0.0(4.0)^a^ HannumAge:−0.01(2.99)^a^ PhenoAge:−0.1(4.8)^a^ GrimAge:−0.1(4.1)^a^ DunedinPoAm:1(0.07)^a^			smoking status, alcohol consumption, BMI, pack years, years of education, physical activity,	Although a positive trend was revealed in GrimEAA after adjustments, all findings and irrespective of confounder adjustments, were non-significant (all *p* > 0.05). Despite this insignificance, the majority estimated association shown a negative relationship in original model, whereas shifted toward a positive trend after adjusting for covariates	**∅**
Markon et al. (2024)	Stress	Psychosocial stress	−0.07(0.08)	HorvathAge, HannumAge, GrimAge, PhenoAge, DunedinPACE age			GrimEAA: β=0.114(SE=0.035, *p* < 0.001) DunnedinPACE: β =0.118(SE=0.031, *p* < 0.001)	age, age squared (i.e., quadratic), sex, smoking status, drinking behavior, BMI, sleep quality, cognitive status	A positive association was found between general psychosocial stress and both GrimAge acceleration and DunedinPACE age acceleration	
Nwanaji et al. (2023)	Stress	PSS	25.9(7.5)	HorvathAge, HannumAge, PhenoAge, GrimAge, SkinBlood Clock, IEAA, EEAA	HorvathAge:74.1(6.9)^b^ HannumAge:77.5(7.6)^b^ PhenoAge: 68.1(8.3)^b^ GrimAge: 68.0 (6.5)^b^ SkinBlood: 75.3 (6.7)^b^	HorvathEAA: *b*=−0.55 (95 % CI [−1.03, −0.77] HannumEAA: *b* =−0.79(95 % CI [−1.31,−0.27], *p* = 0.003) EEAA: *b* =−0.96(95 % CI [−1.58,−0.34], *p* = 0.003) SkinBloodEAA: *b* =−0.52 (95 % CI [−0.95,−0.05]	HorvathEAA: *b*=−0.59(95% CI [−1.08,−0.11], *p* = 0.02) HorvathEAA sensitivity (above 75th percentile stress): *b* = 2.29 (95% CI [0.16,4.41], *p* = 0.04) HannumEAA: *b* =−0.79 (95% CI [−1.31, −0.27], *p* = 0.003) IEAA: *b* =−0.46(95%CI[−0.91,−0.01], *p* = 0.05) EEAA *b* =−0.95(95% CI [−1.57,−0.32, *p* = 0.003] SkinBloodEAA: *b* =−0.50 (95% CI [−0.96,−0.03]	smoking status, alcohol consumption, BMI, pack years, years of education	Baseline analysis revealed an inversed association between stress and epigenetic age acceleration quantified by first-gen, SkinBlood, IEAA, and EEAA. However, a longitudinal sensitive analysis including participants above the 75th percentile for stress suggested a strong positive association between stress and HorvathEAA. All estimates from second-generation clocks displayed positive trends, though they did not reach statistical significance (all *p* > 0.05).	
Skinner et al. (2023)	Stress	Stressful Live Events questionnaire	Low Stress: 1592 High Stress: 2109	PhenoAge, GrimAge, IEAA, EEAA	PhenoAge:57.17(6.99)^b^ GrimAge:57.96(6.98)^b^		GrimEAA: *b* = 0.34 (95%CI [0.08–0.59] EEAA: *b* = 0.47 (95% CI [0.04,0.91]	BMI, sex, genetics, Cell proportions, income, education level, marital status,	A positive association was identified between GrimEAA, EEAA, and stress burden. Statistical power remained constant in the sensitivity analysis, indicating a robust association between GrimAge and health-related outcomes. The association between stress on epigenetic age acceleration was more pronounced in the group with lower social support.	
Suglia et al. (2024)	Stress	Stressor Life Period Composite Score Components	0.0113(1)	HorvathAge, HannumAge, GrimAge, PhenoAGe, DunedinPACE, SkinBloodAge	HorvathAge:58.73(4.36)^b^ HannumAge:62.16(4.11)^b^ PhenoAge:43.44(5.97)^b^ GrimAge:64.57(4.39)^b^ DunnedinPACE:0.97(0.14)^b^ SkinBloodAge:60.3(2.94)^b^		GrimEAA: *b* = 0.808(SE=0.215, *p* < 0.00714) DunnedinPACE: β=0.037, SE=0.007, *p* < 0.00714 SkinBloodEAA: *b* = 0.488, SE=0.156, *p* < 0.00714	sex, race, smoking status	Total life stress score and adult stress score were positively associated with GrimEAA and DunedinPace, regardless of gender. Childhood stressor score was associated with increasing aging among females, whereas not males. Adulthood stressor score was associated with age acceleration in males but not females, revealing the gender-based difference in vulnerability.	
Vetter et al. (2022)	Stress	PSS	2.08 (0.64)	7CpGs, HorvathAge, HannumAge, PhenoAge, GrimAge	HorvathAge:0.03(4.04)^a^ HannumAge:0.01(3.89)^a^ PhenoAge:0.04(5.42)^a^ GrimAge:0.03(3.39)^a^	HorvathEAA:b=−0.290(SE=0.129, p = 0.025) PhenoEAA: b=−0.390(SE=0.174, p = 0.025)		Sex,smoking status, alcohol intake (yes/no), BMI, education, genetic ancestry	Inversed associations between stress and epigenetic aging were identified in two unadjusted models: Horvath Age and PhenoAge	
Zannas et al. (2015)	Stress	Stressful Live Events questionnaire		HorvathAge	−0.13(5.69)^a^		HorvathEAA: *b* = 0.31 (SE=0.11, *p* = 0.0074)	age, sex, BMI, smoking status, alcohol intake. Drug use	A direct association was observed between HorvathEAA and lifetime stress. Stressor type stratified analysis indicated a positive association between personal life stressors and EAA, but not with network stressors and current stress.	
Brown et al. (2017)	Depression	CES-D	Aged-match control: 3866 (<10) Significant Depressive symptoms: 2366 (≥ 10)	LevinAge					The study found that individuals with significant depressive symptoms (CES-D ≥ 10) had a biological age that was, on average, 1.28 years higher than non-depressed individuals (CES-D < 10). Chronological age (CA) showed no significant association with depression.	
Han et al. (2018)	Depression	IDS self-report-30-items	Aged-match control (IDS<14): 319 MDD (IDS≥14):811	Custom MBD-Seq-Based Clock	0(3.58)		*b* = (0.10, *p* = 0.001, depression treated as continuous) *b* = 0.64(*p* = 0.008, MDD vs. control)	Sex, education, BMI, cotinine levels, Alcohol Use, Physical activity, number of chronic diseases	Epigenetic age in individuals with MDD was estimated to have an additional 7.68 months of biological aging relative to chronological age, compared to control. This acceleration was more pronounced in patients concurrently reporting childhood trauma. Findings were replicated using post-mortem brain tissue, demonstrating cross-tissue consistency. Enrichment analysis revealed that genes associated with methylated CpG sites involved in EA mitigate neuronal growth and survival.	
Joshi et al. (2024)	Depression	CES-D 10 items	5.7(6.14)	HorvathAge, HannumAge, PhenoAge, GrimAge	PhenoAge:42.81(12.92) GrimAge:56.32(11.02)	GrimEAA: b = 0.11(95% CI [0.05,0.16], p < 0.05)	GrimEAA: *b* = 0.07(95% CI [0.01,0.13], *p* < 0.05)	Sex, income, number of people living together, chronic disease, poor health behaviors	A positive association between depressive symptoms and GrimEAA was identified in both unadjusted and adjusted model. Although results from other clocks (*n* = 3) were non-significant, all estimated results featured positive direction.	
Liu et al. (2022)	Depression	BDI-II	Baseline: 7.0(8.3) Follow up:5.8(6.4)	HorvathAge HannumAge PhenoAge GrimAge IEAA EEAA			At baseline: HannumEAA: *b* = 0.81 (95% CI [0.23, 1.40], *p* = 0.008), EEAA: *b* = 1.05(95% CI [0.35,1.75], *p* = 0.004) At follow up: PhenoEAA: *b* = 1.33 (95% CI [0.19,2.47], *p* = 0.025) GrimEAA: *b* = 1.00(95% CI [0.024,1.98], *p* = 0.049)	Zygosity, smoking status, cardiovascular disease history, BMI, alcohol consumption, physical activity	The twin effects model, which accounts for the within-pair difference, identified the direct relationship between depression, HannumEAA and EEAA at baseline. At follow up, these results became non-significant, whereas significant and positive associations were observed in all second-generation clocks. Regarding those non-significant findings, persistent and positive associations were observed across time points except for HorvathEAA and IEAA.	
Protsenko et al. (2021)	Depression	IDS-SR	Aged-match control:4.2(3.5) MDD:32.5(8.9)	GrimAge	Aged-match control: −1.01(2.62) MDD:0.63(2.60)	*t*=−3.265 (p = 0.001, Cohen’s d = 0.63)	*F* = 6.095(*p* = 0.015, Cohen’s d = 0.48)	Sex, BMI, smoking status	Primary analysis suggests a median GrimAge acceleration of 2 years in individuals with MDD compared to age-matched controls. This finding remained significant in ANCOVA models after adjusting for covariates (e.g., smoking, sex, BMI). Notably, GrimEAA was not associated with the duration of the current depressive episode nor the overall chronicity of depression	
Robert et al. (2024)	Depression	Baseline: five-items mental Health Index Follow-up: CES-D	Baseline: Probable Depression: 11.8%	HorvathAge HannumAge PhenoAge GrimAge DunedinPoAM DunedinPACE				Age, BMI, race, cell proportions, ancestry, smoking status, alcohol consumption, diet, physical activity	Among six epigenetic clocks analyzed, none demonstrated significant association with probable depression in either cross-sectional or longitudinal analysis. Conflicting trends were observed: cross-sectional analysis generally suggested an inversed relationship, whereas longitudinal analysis suggested a slightly positive trend between EAA (first, second gens, and DunedinPACE) and depression, after adjusting for covariates. These trends persisted when the analysis was restricted to participants retaining depression alone.	**∅**
Simons et al. (2023)	Depression	Mini-MASQ	1.27(0.36)	Cortical BrainAge	54.05(7.15)		*b* = 0.655 (SE=0.367, *p* < 0.1)	loneliness, Brain derived neurotrophic factor, gender, education, linear growth rate, various epigenetic clocks	A positive trend was observed between depression and cortical aging (*b* = 0.655) in a sample of 693, though the result was not statistically significant (*p* < 0.1). The study focused on the effects of depression and loneliness, with multicollinearity between these variables potentially explaining the non-significant result.	**∅**
Sorlí et al. (2024)	Depression	BDI-II	9.0 (0.3)	DunedinPACE	DunedinPACE ranging from 0.66 to 1.53		*b* = 0.003(± 0.001), *p* =	Age, sex, education, BMI, diabetes status, smoking, physical activity, and sleep duration,	The association between depression score and DunedinPACE was only significant in a sex-stratified analysis indicating that greater depressive symptoms in females, but not males were associated with accelerated aging rate.	
Tanifuji et al. (2023)	Depression		MDD: 210 Control:489	HorvathAge HannumAge PhenoAge GrimAge SkinBloodAge				Age, sex	Although none of the model estimation reached statistical significance, all estimated DNAm based EAA pointed toward a positive association in MDD patients. Two EAA measures, HannumEAA (*p* = 0.066) and PhenoEAA (*p* = 0.061) approached threshold of statistical significance. The strongest impact was observed in the PhenoEAA, suggesting an additional 1.25 years of biological age relative to chronological age in MDD, followed by 0.81 and 0.47 in the HannumEAA and GrimEAA, respectively, with basic covariates adjusted	**∅**
Wang et al. (2024)	Depression	CES-D 10 items	1.4(2.0) High depressive symptoms (≥ 4): 556(14%)	HorvathAge HannumAge PhenoAge GrimAge MOPA	HorvathAge:0(6), HannumAge: 0(5.2), PhenoAge: 0(7), GrimAge: 0(4.8), MOPA:0(6)		Depression as continuous:HannumEAA: *b* = 0.14(SE=0.4, *p* < 0.001) PhenoEAA(*b* = 0.20, *p* < 0.001) GrimEAA: *b* = 0.24(SE=0.04, *p* < 0.001) MPAEAA: *b* = 0.19(SE=0.05, *p* < 0.001) High v.s. Low depression: PhenoEAA: *b* = 0.77 (SE=0.31, *p* < 0.05) GrimEAA: *b* = 1.15 (SE=0.19, *p* < 0.001)		Significant associations were observed between depression and all EAA except for HorvathAge. Participants with high depressive symptoms (CES-D ≥ 4) were estimated to have an additional 1.7 years of GrimEAA for males and 0.9 years for females.	
Beach et al. (2022)	Loneliness	UCLA Loneliness	Baseline:3.420 (1.486) Followup:6.730 (1.636)	DunedinPACE	Baseline:1.057(0.143) Followup:1.111(0.142)		*b* = 0.012 (SE = 0.004 *p* < 0.01)	Age, sex, smoking status, alcohol consumption, per capita income	A linear growth in DunedinPACE was observed across time points. After adjusting for covariates, this growth was positively associated with loneliness. The effect size was attenuated but remained significant (*p* < 0.01) after further adjustment for various immune cell types, reflecting an intrinsic DunedinPACE measure.	
Freilich et al. (2024)	Loneliness	UCLA Loneliness	1st wave:4.29 (3.2) 2nd wave:4.97(3.41) 3rd wave:5.12 (3.34)	HorvathAge HannumAgePhenoAge GrimAge DunedinPoAM EAA average	HorvathAge: 65.7(9.6) HannumAge: 54.6(9.2) PhenoAge: 57.5(10.1) GrimAge: 68.1(8.6) DunedinPoAm:1.08(0.09)		GrimEAA: β=0.07(SE=0 02, *p* = 0.003) EAA average: β=0.06 (SE=0.03, *p* = 0.01)	Age, BMI, sex, race,education,smoking status, alcohol consumption	Initial levels of loneliness, measured as the latent intercept across multiple waves, were positively associated with GrimEAA (0.07 SD) and the EAA average (0.06 SD), after adjusting for covariates. Changes in loneliness over time (loneliness slope) were not significantly associated with EAA, except for a weak positive association with GrimEAA (β = 0.08, *p* = 0.04)	
Freilich et al. (2024)	Loneliness	Loneliness three items		HorvathAgeHannumAgePhenoAge, GrimAge DunedinPACE EAA average	HorvathAge:55.5(11.1) HannumAge:42.4(11.5) PhenoAge: 43.6(13.0) GrimAge: 57.1(10.9) DunedinPACE:0.99(0.14)		GrimEAA(β=0.08, SE=0.05, *p* = 0.001) DunedinPACE: β=0.06 (SE=0.02, *p* = 0.02) EAA average: β=0.06(SE=0.03, *p* = 0.01)	Age, BMI, sex, race, education, smoking status, alcohol consumption,	Direct associations were found in GrimAge, DunedinPACE, and EAA average. The adjusted model estimated a 0.08 SD increase of GrimEAA per unit increase in loneliness. The EAA average was a composite outcome variable, taking arithmetic mean and capturing the variance of all employed epigenetic clocks (*n* = 5). The significant and positive effect (*b* = 0.06) in the adjustment model indicates the robustness across 2nd and 3rd generation clocks, confirming its consistency across various measures of epigenetic aging.	
Simons et al. (2023)	Loneliness	UCLA Loneliness	Baseline:1.705(0.738) Follow up:3.362(0.818)	Cortical BrainAge	54.05 (7.15)		*b* = 0.638(SE=0.106, *p* < 0.01)	depression, Brain derived neurotrophic factor, sex education, various epigenetic clocks	Increases in loneliness were positively associated with cortical-based measure of brain epigenetic age (CC-Bd). Net effect of covariates, per score increase in loneliness corresponds to an additional 7.656 months of brain age. This finding remained statistically significant after adjusted for both 2nd and 3rd generation epigenetic clocks as time-varying effect.	

Note: PSS: Perceived Stress Scale; MDD: Major Depressive Disorder; EAA: Epigenetic Age Acceleration; IEAA:Intrinsic Epigenetic Age Acceleration; EEAA: Extrinsic Epigenetic Age Acceleration.

a Mean model residual after regressing epigenetic age on chronological age.

b Mean raw estimated epigenetic age *b*: Unstandardized beta coefficient, β: Standardized beta coefficient Remarks:  model estimated direct association,  model estimated negative associations, ∅ all findings did not reach statistical significance Only model results with *p* < 0.05 are shown in this table; non-significant results are discussed in the main text; original *p* values presented.

Overall, all included studies were from Western countries, with the majority from the United States. Whereas sought for, no eligible anxiety-EAA study was identified; the empirical synthesis, therefore, is limited to stress, depression, and loneliness, with the anxiety domain treated as an important evidence gap. Although exact proportions were not available, few studies enrolled participants younger than 40 years [[39], [40], [41], [42], [43], [44], [45]], and the potential influence of broader age ranges on pooled estimates was subsequently evaluated through sensitivity analyses. Epigenetic age was estimated using peripheral blood-based DNA methylation data across all included studies. One study additionally conducted enrichment analyses using post-mortem brain tissue [40], while another had cortical aging proxy derived from blood-based methylation data [43]. Of the included studies, nine examined the association between stress and EAA [39,[45], [46], [47], [48], [49], [50], [51], [52]], and another ten examined the association between depression and EAA [38,[40], [41], [42], [43], [44],[53], [54], [55], [56]], with aggregated sample sizes of 14,903 and 12,224, respectively. The association between loneliness and EAA was reported in four studies [43,[57], [58], [59]]. No studies investigating anxiety met the inclusion criteria as of the search date.

Stress was primarily measured by the Perceived Stress Scale (PSS), and Depression was primarily measured by the Center for Epidemiological Studies Depression (CES-D) scale. Loneliness was assessed using the UCLA loneliness scale and a three-item loneliness scale. The two scales are found to be similar in terms of quantifying loneliness [57]. Twelve studies concurrently reported findings from both first- and second-generation epigenetic clocks [42,44,[47], [48], [49],^51^,^52^,^54^,[56], [57], [58],^60^]. Findings from third-generation clocks were reported in eight studies [38,42,48,51,[56], [57], [58],^61^]. Furthermore, several studies reported intrinsic EAA (IEAA) and extrinsic EAA (EEAA) concurrently [49,50,55].

### 3.2 Quality assessment

Sample sources were described in all studies, and demographic and lifestyle covariates were adjusted in most. The primary concern was the inadequacy of follow-ups. Overall, sixteen studies were classified as high quality [40,42,43,[45], [46], [47], [48], [49], [50],^52^,^54^,[56], [57], [58],^60^,^61^], five as moderate quality [38,39,41,51,53], and one as low quality [44]. Results of the quality assessments are available in **Appendix B** of the supplementary material.

### 3.3 Findings of stress-EAA

Two studies reported inverse associations (*b* ranging from −0.59 to −0.29) between stress and HorvathEAA [49,52]. Conversely, one study found that greater stressful burden was associated with higher HorvathEAA (*b* = 0.31, SE = 0.11, *p* = 0.007) [45]. Positive trends were also observed in others [48,51], although these associations did not approach statistical significance. Findings related to HannumAge were similarly inconsistent. One study indicated a significant inverse association (*b* = −0.79, 95 % CI [−1.31, −0.27], whereas others [47,48,51,62] found no significant associations between stress and HannumEAA.

Among seven studies analyzing PhenoAge [[46], [47], [48], [49], [50], [51], [52]], significant associations were reported in two [46,52]. Boen et al. (2023) found that higher cumulative stress was associated with greater PhenoEAA (*b* = 0.705, SE = 0.125, *p* < 0.001) and that PhenoEAA was greater in African Americans compared to whites and Asian Americans at baseline. In contrast, one negative association was found in an unadjusted model (*b* = −0.39, SE = 0.17, *p* = 0.03), which became significant after covariate adjustments [52]. As for another second-generation clock, GrimAge, significant findings were reported in four [39,48,50,51]. A pronounced association was observed in an adjusted model in Suglia et al. (*b* = 0.81, SE = 0.22, *p* < 0.007). Additionally, the association between total life stress (i.e., a composite measure of childhood and adulthood stress) and GrimEAA remained statistically significant after adjusting for covariates and was independent of sex. However, childhood stress was associated with accelerated aging only among females, whereas the effects of adulthood stress were pronounced in males and not observed in females. These findings suggest the possibility of sex-specific trajectories linking stress to aging.

Interestingly, social support appeared to attenuate the adverse effects of stress on GrimEAA [50]. However, race-stratified analyses indicated that African American females had a higher risk of accelerated aging under stressful events, regardless of the social support availability [51]. These findings are consistent with racial disparities reported by Boen et al. (2023).

One study examined both continuous and categorical measures of stress and resilience. In unadjusted analyses, higher stress levels were associated with GrimEAA (*b =* 0.24, *p =* 0.02) [39]. However, the association between the reference group and individuals with high stress and low resilience was no longer significant after covariate adjustment.

Findings for EEAA were mixed. Skinner et al. found a positive association between EEAA and stress, while Enwerem et al. indicated an inverse relationship. The remaining studies found no statistically significant associations.

### 3.4 Findings of depression-EAA

Overall, direct associations between EAA and depression were observed in the majority of studies [40,41,[53], [54], [55], [56]], whereas three studies were inconclusive [38,42,43]. One study revealed that individuals with major depressive disorder (MDD), on average, possess an additional 0.64 years (i.e.,7.68 months of bio-age) (measured by methyl-binding domain sequence-based epigenetic age) relative to age-matched controls. Findings were further replicated in an enrichment analysis in post-mortem brain tissue (*n =* 141), revealing overrepresentation of neuronal growth, regulation, and survival pathways [40].

Although positive trends were generally observed, the reported association between depression and HorvathAge was not statistically significant in the literature [42,44,[54], [55], [56]]. Regarding Hannumage, a positive association was identified in one study when depression was modeled as a continuous trait (*b* = 0.14, SE = 0.04, *p* < 0.01), whereas it became non- significant when comparing probable depression CES-D > 4) to age-matched controls [56].

Of the five evaluating PhenoAge [42,44,[54], [55], [56]], one study revealed that a per -unit increase in CSE-D corresponded to an additional 0.20 years of PhenoEAA (*b =* 0.20, p < 0.001) [56]. The study additionally revealed that individuals with high depressive symptoms (CES-D > 4), on average, exhibited an additional 0.77 years of PhenoEAA (*b =* 0.77, SE = 0.31, p < 0.05) [56]. However, other analyses failed to reject the null hypothesis, despite the estimated trends all being positive. When comparing diagnosed MDD patients with age-matched controls, a marginally significant, positive trend was identified by Tanifuji et al., suggesting an additional 1.25 years of EAA in MDD patients (*p* = 0.06).

Four studies reported significant, positive associations between depressive symptoms and EAA calculated with GrimAge [41,[54], [55], [56]], with the standardized effect, β, ranging from 0.06 to 0.10 in non-clinical populations. One study found that each additional CES-D score corresponded to an increase of GrimEAA by 0.24 points (*b* = 0.24, SE = 0.03, *p* < 0.001) [56]. This effect was subsequently found in a group comparison between individuals categorized as having high depressive symptoms (CES-D > 4) and non-depressed individuals (*b* = 1.15, SE = 0.19, *p* < 0.001). The strongest association (*F*_MDD_ = 6.10, *p* = 0.02, Cohen’s *d* = 0.48) was observed when comparing MDD with aged match controls [41]. Similar patterns were also observed in a study analyzing EAA (GrimAge) in MDD, but the results were non-significant [44].

### 3.5 Findings of loneliness-EAA

Direct associations were observed in all studies [43,57,58,61]. Simons et al. observed that a within-person increase of the UCLA loneliness scale by one unit was associated with an additional 0.638 years of cortical aging (*b* = 0.638, SE = 0.106, *p* < 0.01) over time. In a longitudinal study, linear growth in DunedinPACE across time points was positively associated with loneliness (*b* = 0.012, SE = 0.004, *p* < 0.01), suggesting a potential role for loneliness in accelerating the aging pace [61]. Two studies conducted by the same group of researchers, but with different cohorts analyzed, yielded consistent results [57,58]. Both studies reported significant, positive loneliness-GrimEAA associations and an EAA average association, defined as the arithmetic mean accounting for the variance of the five epigenetic clocks included in their studies. The remaining associations exhibited positive trends, whereas none were significant.

### 3.6 Meta-analysis

**Fig. 2:**
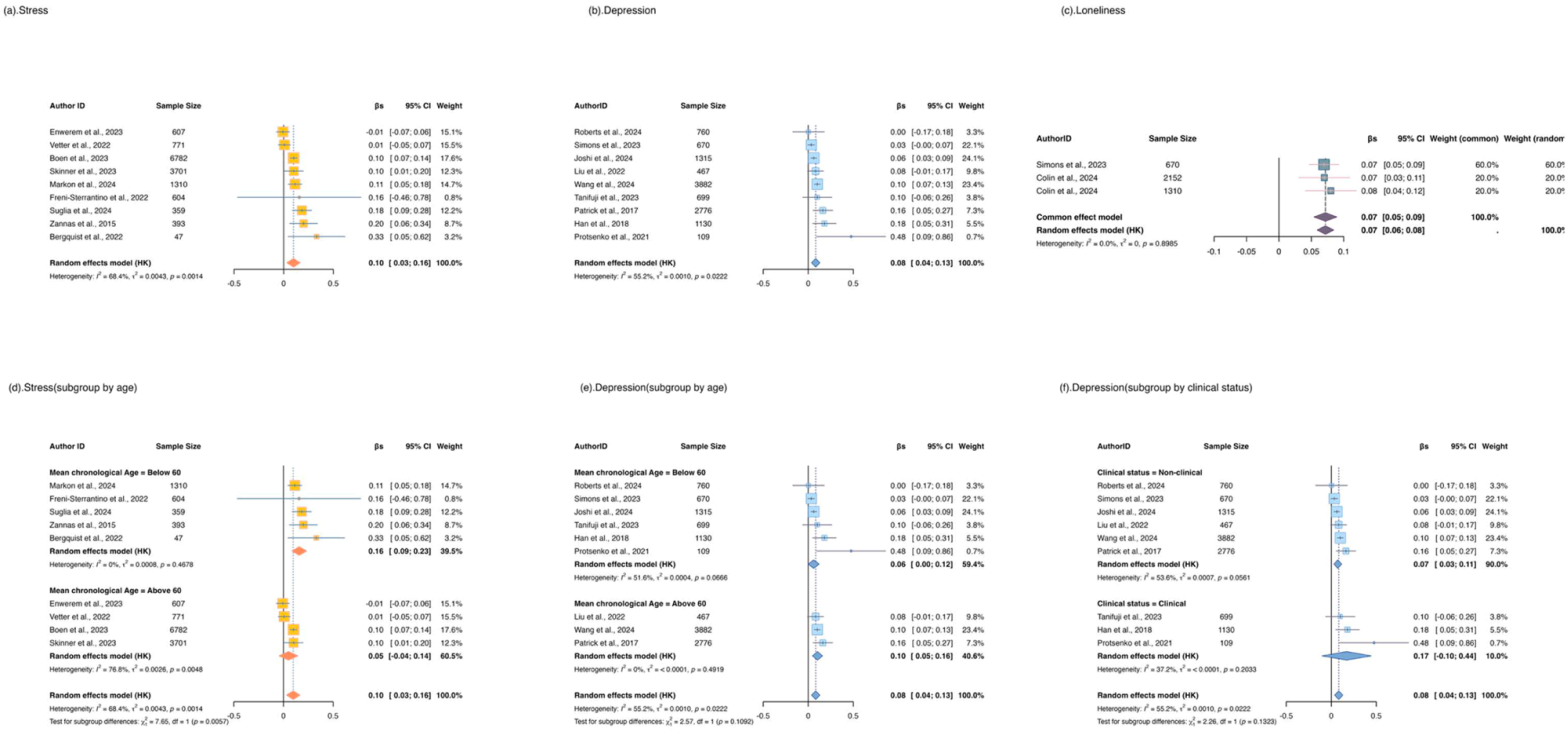
Forests plots summarizing the primary meta-analyses. (a) Overall association between psychosocial stress and epigenetic age acceleration. (b) Overall association between depression and epigenetic age acceleration. (c) Overall association between loneliness and epigenetic age acceleration. (d) Association between psychosocial stress and epigenetic age acceleration stratified by mean chronological age. (e) Association between depression and epigenetic age acceleration stratified by mean chronological age. (f) Association between depression and epigenetic age acceleration stratified by clinical status. Reference categories: mean chronological age < 60 years; clinical status = non-clinical. Effect sizes were pooled using a random-effects model (τ² estimated via restricted maximum likelihood). Group confidence intervals were estimated using the Hartung-Knapp method.

The results of the primary meta-analyses are presented as a forest plot (Fig. 2). Random-effects models revealed that greater stress (Fig. 2**a**), depression (Fig. 2**b**), and loneliness (Fig. 2**c**) were all associated with higher EAA. For each SD increase in depression (*β* = 0.08, 95 % CI [0.04, 0.13] and loneliness (*β* = 0.07, 95 % CI [0.05, 0.09], there was a corresponding additional 0.08 and 0.07 SD increase in EAA, respectively. The pronounced effect was observed for stress (*β* = 0.10, 95 % CI [0.03, 0.16]. Sensitivity analysis excluding studies in (i). which standardized effect sizes were derived from approximated SD_EAA_, (ii). studies enrolling younger participants despite a mean cohort age ≥ 40 years, (iii). and longitudinal aggregated estimates produced estimates that were statistically significant and robust to the primary analysis (**Supplemental Tables 1–2**).

#### 3.6.1 Clock-specific meta-analysis

Clock-specific meta-analyses (**Supplemental Figures S1-S3**) further revealed that both depression (*β* = 0.08, *p =* 0.005) and loneliness (*β* = 0.08, *p* < 0.001) were positively associated with GrimAge. This was followed by a positive association observed between loneliness and DunedinPACE (*β* = 0.06, 95 % CI [0.04, 0.08]. Despite directionally positive, the remaining estimated effects were not statistically significant across domains and clock types.

#### 3.6.2 Post-hoc analyses evaluating heterogeneity

Substantial between-study heterogeneity was observed in both primary meta-analyses (stress-EAA: *I^2^* = 68.4 % (p = 0.001); depression-EAA: *I^2^* = 55.2 % (p = 0.022). We first performed influential case analyses to identify potential outliers. Although several studies approached the threshold, none were classified as influential cases **(Supplemental Figures S4-S7)**. Leave-one-out refitting did not meaningfully alter the group effect sizes or the heterogeneity y in either meta-analysis **(Supplemental Figures S8, S9)**.

Exploratory GOSH analyses subsequently revealed a dominant cluster of pooled effects for both primary meta-analyses, indicating general stability across a wide range of subset combinations. GOSH diagnostics identified several studies that disproportionately contributed to heterogeneity, despite not being flagged by conventional leave- one-out diagnostics (**Supplemental Figures S10-S11**). GOSH-pruned models were therefore treated as secondary sensitivity analyses. After excluding GOSH-flagged studies, effects remained statistically significant and directionally consistent, while heterogeneity was reduced (stress-EAA: *β* = 0.12, 95 % CI [0.08, 0.16], *I^2^* = 5.1 %; depression-EAA: *β* = 0.10, 95 % CI [0.05, 0.14], *I*^2^ = 41.8 %) (Supplemental Tables 1–2).

#### 3.6.3 Meta-regression

Meta-regressions were performed using mixed-effects models, with two subgroups (e.g., mean chronological age (*Ref. below 60*), clinical status (*Ref: clinical depression*) dichotomized as dummy variables. Results are presented in the Fig. 2**c-**2**e** and **Supplemental Tables** [3–5]. The model first revealed that the stress-EAA association was pronounced in individuals aged 60 years and younger (*β_0_* = 0.17 (95 % CI [0.09, 0.24], *p* = 0.02) but significantly attenuated among those aged 60 years and older (*β_1_* = −0.12, 95 % CI [−0.23, −0.02]. Although heterogeneity remained, the moderator alone explained 51.3 % of the variance.

Two additional meta-regressions were performed to investigate the group effect on the depression-EAA association. Overall, the depression-EAA relationship was pronounced in individuals aged above 60 years (β_1_ = 0.05, 95 % CI [−0.02, 0.11] and in MDD patients (β_1_ = 0.10, 95 % CI [−0.04, 0.24] compared to the respective reference group. While both variables reduced residual heterogeneity to random sample error, neither moderator was statistically significant (all *p* > 0.05). These results are consistent with subgroup forest plots (Fig. 3c, 3d, 3e), but *p-values* are considered more robust in meta-regressions.

#### 3.6.4 Assessment of publication bias

Overall, no clear evidence of publication bias was observed for the reported domains. Contour-enhanced funnel plots (**Supplemental figures S12, S13**) indicated that estimated effects were symmetrically distributed around the pooled effect size. This was complemented by two formal Egger’s tests. The intercept was estimated at *b*_0_ = 1.00 (*t* = 0.90, 95 % CI [−1.2, 3.21], p = 0.40) for stress-EAA and *b_0_* = 1.16 (*t =* 1.50, 95 % CI [−0.35, 2.67], p = 0.18) for depression-EAA meta-analysis, with both testing failing to reject the null hypothesis of symmetry.

Trim-and-fill analyses imputed three studies in the stress-EAA and two in the depression-EAA analysis (**Supplemental figures S1-S15**). Adjusted estimates were directionally consistent with the primary findings. Additional sensitivity analyses excluding GOSH diagnostics-flagged outliers also produced comparable effects (**Supplemental Tables 1–2**). *P-*curve analyses incorporated *K =* 6 and *K =* 5 significant effects from stress and depression meta-analyses, respectively; detailed estimates are presented in **Supplemental Tables 6 and 7**. Since these analyses were conducted near the minimum threshold of K and the primary models showed moderate to substantial heterogeneity, both trim-and-fill and p-curve analyses were interpreted as illustrative rather than confirmatory.

## 4 Discussion

### 4.1 Summary of findings

Psychological adversities (PA), through their systemic effects on health, may be embedded in biological aging trajectories. A quantitative synthesis of their effects becomes necessary for policy development and precision geromedicine. This review, to the best our best knowledge, is the first to aggregate evidence from literature examining the association between PA and epigenetic age acceleration (EAA) in midlife and above.

Across domains, significant associations were primarily observed for second-generation clocks (e.g., PhenoAge and GrimAge), while most first-generation clocks (HorvathAge and HannumAge) estimates were not significant despite generally positive trends. This heterogeneity likely reflects differences in training purposes such that first-generation clocks are designated to predict chronological age, while second-generation clocks are optimized to forecast health span and mortality [34], and have been shown to associate more strongly with health outcomes [33]. Direct associations were also observed for those capturing biological aspects of aging (e.g., IEAA, EEAA). IEAA represents residuals of regressing HorvarthAge on chronological age, adjusted for ad-hoc cell counts [63], and EEAA refers to residuals of regressing HannumAge on chronological age, adjusted for plasma blasts, naïve T cells, and exhausted T cell frequencies [18], all of which extend beyond pure cellular aging, accounting for immunosenescence. These observations indicate that both cellular and immune-related aging trajectories may be intertwined with stress, loneliness, and depression.

Preliminary evidence suggests that stress-EAA associations may differ by racialized social experience, with some studies indicating stronger associations among African American participants. These differences may reflect uneven exposure to persistent discrimination and structural disadvantage among racial minorities [64], which, in turn, are linked to dysregulated stress physiology, inflammation, and pathways implicated in biological aging [65]. Importantly, most available data derive from U.S.-based cohorts composed substantially of individuals of African or European descent and, in several large cohorts, predominantly older women. However, evidence from other geographic contexts, including East Asia, remains scarce.

Our meta-analyses revealed modest but consistent associations of psychosocial stress (*β* = 0.10), depression (*β* = 0.08), and loneliness (*β* = 0.07) with higher EAA. Although prior research has linked modest EAA increases to comorbidity and cognitive phenotypes [[66], [67], [68], [69]], the clinical relevance of the present pooled effects remains uncertain, especially given heterogeneity in clock type and measurement instruments.

Notably, the pooled estimates should not be interpreted as independent effects. Loneliness, depression, and stress are behaviourally and epidemiologically interrelated and commonly co-exist. For instance, loneliness may contribute to depressive symptoms and perceived stress [70,71], while depression may stimulate social withdrawal and subjective loneliness [72]. Among studies, all except Simons et al. [43] evaluated only one psychological domain at a time, without adjustment for other potentially interrelated domains. Consequently, the meta-analytical evidence cannot distinguish domain-specific associations from shared psychological burden or potential mediation among these patterns. Future longitudinal and intervention studies incorporating dimensional domains are promising for determining whether targeting these adversities can meaningfully alter EAA trajectories, clarifying these factors as a modifiable risk in biological aging.

### 4.2 Potential mechanism

The following is inferred from broader geroscience, psychology, and neuroendocrine literature for hypothesis-generating. Psychosocial stress, depression, and loneliness have been linked to the hypothalamus-pituitary-adrenal (HPA) axis dysregulation in existing literature, often demonstrating sustained elevations of circulating cortisol [[73], [74], [75], [76], [77]]. Cortisol exerts its biological effects primarily through two DNA-binding complexes: mineralocorticoid receptors (MRs) and glucocorticoid receptors (GRs). Excessive and/or prolonged exposure to cortisol has been associated with vascular dysfunction and oxidative stress, endothelial aging, impaired glucocorticoid sensitivity (i.e., dysregulated negative feedback loop of HPA axis), and low-grade sterile inflammation (long refs), all of which are plausible pathways contributing to biological aging [[78], [79], [80], [81], [82], [83]]. Alternative pathways, including health behaviors, sleep, and medication use, may also contribute to those associations captured by second-generation clocks.

From an evolutionary perspective, not all stressors are necessarily detrimental. Activation of the *fight-or-flight* response may promote survival [84,85], and moderate stress exposure can exert hormetic effects that potentially mitigate age-associated decline [86,87]. This framework applies to humans. Greater psychological resilience is often observed among older adults as an indicator of HA [74,88]. Such resilience is, in turn, associated with adaptive mechanisms, including robust endocrine regulation, rapid recovery from crisis, and effective adaptation [74].

However, alternative explanations should be highlighted. PA may become biologically embedded through cumulative exposure and the pathway processes across the life span. In view of the life course model [89], the measured EAA in midlife or older age rather reflects accumulated exposure over decades than a consequence of contemporary psychological events (i.e., stress, depression, loneliness). Likewise, the meta-regression- inferred stress-EAA age differences may reflect psychological resilience, cohort differences, or survivor bias. Such findings should therefore be interpreted as hypothesis-generating rather than definitive evidence.

### 4.3 Limitations

The strengths of this review include the concurrent evaluation of multiple common psychological adversities in midlife and older adults, along with comprehensive sensitivity analyses that supported the robustness of the findings. However, several limitations warrant acknowledgment. First, the literature search was restricted to English-language, which may have introduced language bias. The current evidence base may have underrepresented non-Western settings and may reflect systematic differences in education, healthcare access, and sociocultural stressors rather than generalizability alone. Second, exposure measurement varied across domains and studies, including different instruments, reference periods, cutoffs, and distinctions between diagnosed MDD and elevated depressive symptoms (i.e., probable depression). Relatedly, the evaluated psychological domains were not mutually exclusive. As only aggregate published estimates were available, we could not quantify overlap between stress, depression, and loneliness. Therefore, the pooled estimates should be viewed as domain-specific associations, rather than evidence revealing that each condition has an independent effect on EAA. Future research may consider mutually adjusted or mediation analysis to disentangle potential pathways underlying these mechanisms. Third, the limited number of studies (K) may have reduced the statistical power, especially when pooling the loneliness-EAA estimates. Likewise, the meta-regressions and publication bias testing were rather exploratory and constrained by the limited K. Fourth, effect-size standardizations based on approximated SD_EAA_ may have distorted variance estimates and weights, despite sensitivity analyses demonstrating that primary estimates were robust to these approximations. Fifth, all estimates were based on blood-derived DNA methylation data; tissue-specific and post-mortem findings should be interpreted as illustrative. Additionally, NOS scores primarily assess reporting quality and confounder handling but do not establish causal validity; future reviews may benefit from complementary tools, such as ROBINS-I [90] when intervention studies suffice. Finally, most included studies were cross-sectional, and bidirectionality is plausible (**Appendix Table 4**). The present synthesis does not distinguish PA → EAA from EAA → PA; longitudinal, genetically informed, interventional designs are necessary to strengthen both temporal and causal inference.

## 5 Conclusions

This review study suggests that psychosocial stress, depressive symptoms/depression, and loneliness are each associated with accelerated aging from midlife onward. The observed effects were modest, domain-specific, and most consistent for second-generation epigenetic clocks. Given the small number of studies, methodological heterogeneity, and overlap among psychological adversities, these findings should be considered preliminary. Future longitudinal and interventional studies across diverse populations, with concomitant evaluation of related domains, are warranted to clarify shared, bidirectional and causal pathways and determine whether intervention can meaningfully decelerate aging trajectories among at-risk older adults.

## Funding sources

This work was supported by grants from the National Medical Research Council of Singapore (grant nos. NMRC/TA/0053/2016 and NMRC/CSA/INV/0009/2022) awarded to the corresponding author, Dr. Lei Feng. The funders had no role in the study design; data collection, analysis, or interpretation; writing of the report; or the decision to submit the article for publication.

## Declaration of the use of generative AI and AI-assisted technologies in scientific writing and in figures, images and artwork

No generative AI was used in the writing of this manuscript or in the preparation of figures, images throughout.

## Ethical statement

This study is a review study based exclusively on previously published and publicly available data. Therefore, ethical approval and informed consent were not required.

## Data statement

The data supporting the findings of this study are derived from publicly available published studies and are available from the corresponding publications cited in the manuscript.

## CRediT authorship contribution statement

**Jiuyu Guo:** Writing – review & editing, Writing – original draft, Validation, Software, Project administration, Methodology, Investigation, Formal analysis, Data curation, Conceptualization. **Jiatong Shan:** Writing – review & editing, Data curation. **Kaisy Xinhong Ye:** Writing – review & editing, Methodology, Data curation. **Wei Liang:** Writing – review & editing, Data curation. **Yanyu Wang:** Writing – review & editing. **Eng-Tat Ang:** Writing – review & editing. **Tih-Shih Lee:** Writing – review & editing. **John Suckling:** Writing – review & editing. **Andrea B. Maier:** Writing – review & editing. **Lei Feng:** Writing – review & editing, Supervision, Project administration, Methodology, Funding acquisition, Data curation.

## Declaration of competing interest

The authors declare no known competing interests or personal relationships that could have appeared to influence the work reported in this paper.

## Appendix Supplementary materials

Image, application 1
