# Supplementary material for: Psychological adversities and epigenetic ageing in midlife and older age: A systematic review and meta-analysis
Source: J Prev Alzheimers Dis. 2026 Aug 7;13(9):100650. doi: 10.1016/j.tjpad.2026.100650 (PMC13476511; doi:10.1016/j.tjpad.2026.100650)
Supplement: Supplementary file 1 [file mmc1.docx]

SUPPLEMENTARY MATERIALS

Results

*Meta-analysis by epigenetic clocks type*

[Supplemental Figure S1. Forest plot summary of clock type specific meta-analysis (stress)	2](#_Toc219992099)

[Supplemental Figure S2. Forest plot summary of clock type specific meta-analysis (depression)	3](#_Toc219992100)

[Supplemental Figure S3. Forest plot summary of clock type specific analysis (Loneliness	4](#_Toc219992101)

[.	4](#_Toc219992102)

*Influential case analysis*

[Supplemental Figure S4. Influential Case Analysis on Stress Meta-Analysis (Leave-one-out method with Random Effect Model)	5](#_Toc219992103)

[Supplemental Figure S5. Influential Case Analysis on depression Meta-Analysis (Leaving-one-out method with Random Effect Model)	6](#_Toc219992104)

[Supplemental Figure S6. Influence diagnostics for each study included in the meta-analysis of stress and epigenetic age acceleration.	7](#_Toc219992105)

[Supplemental Figure S7. Influence diagnostics for each study included in the meta-analysis of depression and epigenetic age acceleration.	8](#_Toc219992106)

[Supplemental Figure S8. Leave-one-out sensitive *forest plot (stress)*	9](#_Toc219992107)

[Supplemental Figure S9. Leave-one-out sensitive forest plot *(Depression)*	10](#_Toc219992108)

*Publication bias assessment*

[Supplemental Figure S12. Contour-enhanced funnel plot (Stress)	13](#_Toc219992109)

[Supplemental Figure S13. Contour-enhanced funnel plot (Depression)	14](#_Toc219992110)

[Supplemental Figure S14. Contour-enhanced funnel plot (Stress Trim-and-fill)	15](#_Toc219992111)

[Supplemental Figure S15. Contour-enhanced funnel plot (Depression Trim-and-fill)	16](#_Toc219992112)

*Table summary of sensitives analyses*

[Supplemental Table 1. Summary of sensitivity analyses of stress-EAA meta-analysis	17](#_Toc219992113)

[Supplemental Table 2. Summary of sensitivity analyses of Depression-EAA meta-analysis	18](#_Toc219992114)

*Meta-regression*

[Supplemental Table 3. Summary of Mixed-Effects Meta-Regression (Stress)	19](#_Toc219992115)

[Supplemental Table 4. Summary of Mixed-Effects Meta-Regression (Depression subgroup1)	21](#_Toc219992116)

[Supplemental Table 5. Summary of Mixed-Effects Meta-Regression (Depression subgroup2)	23](#_Toc219992117)

[Supplemental Table 6. P-curve analysis (stress)	25](#_Toc219992118)

[Supplemental Table 7. *P*-curve analysis (Depression)	25](#_Toc219992119)

Methods

[Appendix Table 1. Search Strategy	26](#_Toc219992120)

[Appendix Table 2. Table of Characteristics of Excluded Studies	27](#_Toc219992121)

[Appendix A. NEWCASTLE-OTTAWA SCALE (NOS)	29](#_Toc219992122)

[Appendix B. Summary of Study Quality assessment	31](#_Toc219992123)

**Supplemental Figure S1. Forest plot summary of clock type specific meta-analysis (stress)** (a). Estimated association between stress and HorvathEAA; (b). Estimated association between stress and HannumEAA; (c). Estimated association between stress and SkinBloodEAA; (d). Estimated association between stress and PhenoEAA; (e). Estimated association between stress and GrimEAA; (f). Estimated association between stress and DunedinPACE. Effect sizes pooled using random effects model. τ2 estimated using REML estimator; Confidence intervals pooled using Hartung-Knapp method.

## **Supplemental Figure S2.** Forest plot summary of clock type specific meta-analysis (depression)

(a). Estimated association between depression and HorvathEAA; (b). Estimated association between depression and HannumEAA; (c). Estimated association between depression and PhenoEAA; (e). Estimated association between depression and GrimEAA; Effect sizes pooled using random effects model. τ^2^ estimated using REML estimator; Confidence intervals pooled using Hartung-Knapp method

**Supplemental Figure S3**. Forest plot summary of clock type specific analysis (Loneliness) (a). Estimated association between loneliness and HorvathEAA; (b). Estimated association between loneliness and HannumEAA; (c). Estimated association between stress and PhenoEAA; (d). Estimated association between stress and GrimEAA; (e). Estimated association between stress and DunedinPACE. Effect sizes pooled using random effects model. τ^2^ estimated using REML estimator; Confidence intervals pooled using Hartung-Knapp method

## .

## **Supplemental Figure S4.** Influential Case Analysis on Stress Meta-Analysis (Leave-one-out method with Random Effect Model)

**Supplemental Figure S5.** Influential Case Analysis on depression Meta-Analysis (Leaving-one-out method with Random Effect Model)

## **Supplemental Figure S6.** Influence diagnostics for each study included in the meta-analysis of stress and epigenetic age acceleration.

## **Supplemental Figure S7.** Influence diagnostics for each study included in the meta-analysis of depression and epigenetic age acceleration.

## **Supplemental Figure S8.** Leave-one-out sensitive *forest plot (stress)*

## **Supplemental Figure S9.** Leave-one-out sensitive forest plot *(Depression)*

 **Supplemental Figure S10.** GOSH diagnostics for Stress-EAA meta-analysis
Panels show clustering of pooled effect sizes and heterogeneity across all possible study subsets using K-means, DBSCAN, and Gaussian mixture models, along with subset distributions after exclusion of potential outliers.

 **Supplemental Figure S11.** GOSH diagnostics for Depression-EAA meta-analysis
Panels show clustering of pooled effect sizes and heterogeneity across all possible study subsets using K-means, DBSCAN, and Gaussian mixture models, along with subset distributions after exclusion of potential outliers

## **Supplemental Figure S12.** Contour-enhanced funnel plot (Stress)

## **Supplemental Figure S13.** Contour-enhanced funnel plot (Depression)

## **Supplemental Figure S14.** Contour-enhanced funnel plot (Stress Trim-and-fill)

## **Supplemental Figure S15.** Contour-enhanced funnel plot (Depression Trim-and-fill)

| Supplemental Table 1. Summary of sensitivity analyses of stress-EAA meta-analysis | | | | | |
| --- | --- | --- | --- | --- | --- |
| Analysis | ***g*** | **95% CI** | ***p*** | **I^2^** | **95% CI** |
| Main analysis | 0.10 | 0.03, 0.16 | 0.009 | 68.4% | 36.6, 84.2 |
| Analysis | ***g*** | **95% CI** | ***p*** | **I^2^** | **95% CI** |
| Potential Infl. cases excluded^1^ | 0.12 | 0.08, 0.16 | < 0.001 | 5.1% | 0, 72.3 |
| Analysis | ***g*** | **95% CI** | ***p*** | **I^2^** | **95% CI** |
| Study SD_EAA_ approximated excluded^2^ | 0.11 | 0.01, 0.20 | 0.03 | 62.0% | 7.4, 84.4 |
| Analysis | *g* | 95% CI | *p* | I^2^ | 95% CI |
| Trim & fill analysis^3^ | 0.07 | 0.00, 0.14 | 0.06 | 71% | 47.9, 83.9 |
| Analysis | ***g*** | **95% CI** | ***p*** | **I^2^** | **95% CI** |
| Trim & fill analysis with Potential Infl.cases excluded^4^ | 0.10 | 0.07, 0.14 | < 0.001 | 36.1% | 0, 69.5 |
| Analysis | ***g*** | **95% CI** | ***p*** | **I^2^** | **95% CI** |
| Age-restricted analysis^5^ | 0.08 | 0.02, 0.14 | 0.03 | 69.4% | 32.7, 86.1 |
| Analysis | ***g*** | **95% CI** | ***p*** | **I^2^** | **95% CI** |
| Longitudinal estimates excluded^6^ | 0.12 | 0.04, 0.20 | 0.01 | 63.2% | 16.6, 83.8 |
| Note: ^1^ removed as potential outliers identified in GOSH diagnostics: Enwerem et al., Vetter et al. ^2^ removed as studies whose standardized effects sizes conversion were based on approximated standard deviations of epigenetic age acceleration: Enwerem et al., Suglia et al., Skinner et al. ^3^ Trim and fill analysis with three imputed studies: Suglia et al., Zannas et al., Bergquist et al. ^4^ Trim and fill analysis with ^1^ removed  ^5^ removed as studies who enrolled participants below 40 years of age as a strict age-range analysis: Bergquist et al., Zannas et al.  ^6^ removed as studies with longitudinal design and/or longitudinal estimates were approximated: Enwerem et al., Markon et al.  Results rounded into nearest two decimals where appropriate | | | | | |

| Supplemental Table 2. Summary of sensitivity analyses of Depression-EAA meta-analysis | | | | | |
| --- | --- | --- | --- | --- | --- |
| Analysis | ***g*** | **95% CI** | ***p*** | **I^2^** | **95% CI** |
| Main analysis | 0.08 | 0.04, 0.13 | 0.003 | 55.2% | 55.4, 78.8 |
| Analysis | ***g*** | **95% CI** | ***p*** | **I^2^** | **95% CI** |
| Potential Infl. cases excluded^1^ | 0.09 | 0.05, 0.14 | 0.002 | 41.8% | 0, 74.3 |
| Analysis | ***g*** | **95% CI** | ***p*** | **I^2^** | **95% CI** |
| Trim & fill analysis^2^ | 0.07 | 0.02, 0.12 | 0.008 | 60.1% | 22.6, 79.4 |
| Analysis | ***g*** | **95% CI** | ***p*** | **I^2^** | **95% CI** |
| Trim & fill analysis with Potential Infl. cases excluded^3^ | 0.08 | 0.04, 0.13 | 0.002 | 51.1% | 0, 76.2 |
| Analysis | ***g*** | **95% CI** | ***p*** | **I^2^** | **95% CI** |
| Age-restricted analysis^4^ | 0.09 | 0.04, 0.14 | 0.01 | 40.4% | 0, 79.8 |
| Analysis | ***g*** | **95% CI** | ***p*** | **I^2^** | **95% CI** |
| Longitude. design excluded^5^ | 0.11 | 0.04, 0.18 | <0.001 | 55.1% | 0, 82.0 |
| Note: ^1^ removed as potential outliers identified in GOSH diagnostics: Simons et al., Wang et al. ^2^ Trim and fill analysis with two imputed studies: Han et al., Protsenko et al.  ^3^ Trim and fill analysis with ^1^ removed *a priori* ^4^ removed as studies enrolling participants below 40 years of age as a strict age-range analysis: Han et al., Protsenko et al., Roberts et al., Simons et al., Tanifuji et al.  ^5^ removed as studies with longitudinal design and/or longitudinal estimates were approximated: Liu et al., Simons et al., Roberts et al. Results rounded into the nearest two decimals where appropriate | | | | | |

## **Supplemental Table 3.** Summary of Mixed-Effects Meta-Regression (Stress)

| *Coefficients* \| \| \| \| \| \|; \| \| \| \| \| 95% Confidence Interval \|; \| Estimate \| Standard Error \| *z* \| *p* \| Lower \| Upper; Intercept \| 0.1660 \| 0.0333 \| 5.0350 \| 0.0015 \| 0.0874 \| 0.24471; Age Above 60 \| -0.1155 \| 0.0415 \| -2.9942 \| 0.0273 \| -0.2317 \| -0.0173; Note: Wald Test. \| \| \| \| \| \|; Dummy Variable: *I* Age cutoff: *I* = 0(Mean below 60), *I* =1(Mean Above 60) \| \| \| \| \| \|; *R^2^**: Amount of heterogeneity accounted for by the model is *R^2*^* = 51.33% \| \| \| \| \| \| *Statistical Tests* | | | |
| --- | --- | --- | --- |
|  | Q | df | *p* |
| Test of Coefficient | 7.7339 | 1 | 0.0273 |
| Test of Residual Heterogeneity | 16.4725 | 7 | 0.0211 |
| Note: Mixed-Effects Model fitted to K = 9 studies | | | |
| τ^2^ Estimator: Restricted Maximum Likelihood (REML) | | | |

| *Residual Heterogeneity Estimates* | | | |
| --- | --- | --- | --- |
|  |  | 95% Confidence Interval | |
| Parameter | Estimate | Lower | Upper |
| τ^2^ | 0.0021 | 0.000 | 0.0135 |
| τ | 0.0458 | 0.0102 | 0.1162 |
| I^2^ | 56.00% | 0.000 | 89.1262 |
| H^2^ | 2.27 | 1.000 | 9.1964 |

## **Supplemental Table 4.** Summary of Mixed-Effects Meta-Regression (Depression subgroup1)

| *Coefficients* | | | | | | |
| --- | --- | --- | --- | --- | --- | --- |
|  |  |  |  |  | 95% Confidence Interval | |
|  | Estimate | Standard Error | *z* | *p* | Lower | Upper |
| Intercept | 0.0562 | 0.0161 | 2.7198 | 0.0101 | 0.0181 | 0.0942 |
| Age Above 60 | 0.0467 | 0.0262 | 1.6488 | 0.1178 | -0.0153 | 0.1087 |
| Note: Wald Test. | | | | | | |
| Dummy Variable: *I* Age cutoff: *I* = 0(Mean below 60), *I* =1(Mean Above 60) | | | | | | |
| *R^2^**: Amount of heterogeneity accounted for by the model is *R^2*^* = 94.32% | | | | | | |
|  | | | | | | |

| *Statistical Tests* | | | |
| --- | --- | --- | --- |
|  | Q | df | *p* |
| Test of Coefficient | 3.1761 | 1 | 0.1093 |
| Test of Residual Heterogeneity | 11.7430 | 7 | 0.1179 |
| Note: Mixed-Effects Model fitted to K = 9 studies | | | |
| τ^2^ Estimator: Restricted Maximum Likelihood (REML) | | | |

| *Residual Heterogeneity Estimates* | | | |
| --- | --- | --- | --- |
|  |  | 95% Confidence Interval | |
| Parameter | Estimate | Lower | Upper |
| τ^2^ | 0.0001 | 0.000 | 0.0651 |
| τ | 0.0074 | 0.000 | 0.2552 |
| I^2^ | 4.19% | 0.000 | 98.10% |
| H^2^ | 1.04 | 1.000 | 52.56 |

## **Supplemental Table 5.** Summary of Mixed-Effects Meta-Regression (Depression subgroup2)

| *Coefficients* | | | | | | |
| --- | --- | --- | --- | --- | --- | --- |
|  |  |  |  |  | 95% Confidence Interval | |
|  | Estimate | Standard Error | *z* | *p* | Lower | Upper |
| Intercept | 0.0721 | 0.0175 | 3.9739 | 0.0054 | 0.0306 | 0.1135 |
| Clinical Depression | 0.0995 | 0.0599 | 1.6399 | 0.1450 | -0.0423 | 0.2412 |
| Note: Wald Test. | | | | | | |
| Dummy Variable: *I* Mental burden: *I* = 0{subclinical}, *I* =1{clinical depression/MDD} | | | | | | |
| *R^2^**: Amount of heterogeneity accounted for by the model is *R^2*^* = 28.19% | | | | | | |

| *Statistical Tests* | | | |
| --- | --- | --- | --- |
|  | Q | df | *p* |
| Test of Coefficient | 2.75 | 1 | 0.1410 |
| Test of Residual Heterogeneity | 13.96 | 7 | 0.0519 |
| Note: Mixed-Effects Model fitted to K = 9 studies | | | |
| τ^2^ Estimator: Restricted Maximum Likelihood (REML) | | | |

| *Residual Heterogeneity Estimates* | | | |
| --- | --- | --- | --- |
|  |  | 95% Confidence Interval | |
| Parameter | Estimate | Lower | Upper |
| τ^2^ | 0.0007 | 0.000 | 0.0343 |
| τ | 0.0264 | 0.000 | 0.1853 |
| I^2^ | 45.50% | 0.000 | 97.6261 |
| H^2^ | 1.83 | 1.000 | 42.1252 |

## **Supplemental Table 6.** P-curve analysis (stress)

|  |  | **Full Curve** | | **Half Curve** | | **Evidential Value** | |  |
| --- | --- | --- | --- | --- | --- | --- | --- | --- |
|  | *p*_Binomia_*_l_* | *z*_Full_ | *p*_Full_ | *z_Ha_*_lf_ | *p*_Half_ | Present | Absent | $d ̂$ |
| Right-Skewness Test | **0.109** | -4.314 | **<0.001** | -3.777 | **<0.001** | Yes | No | 0.145 |
| Flatness Test | 0.867 | 2.377 | 0.991 | 4.128 | >0.999 | Yes | No | 0.145 |
| Note: $d ̂$: p-curve’s estimate of true effect size | | | | | | | | |
| Binomial test: divides p-values into bins (>.025, <.025) | | | | | | | | |
| Half-Curve: p values within the range of 0 to .025 Full-Curve: within the entire range of 0 to .05 | | | | | | | | |

## **Supplemental Table 7.** *P*-curve analysis (Depression)

|  |  | **Full Curve** | | **Half Curve** | | **Evidential Value** | |  |
| --- | --- | --- | --- | --- | --- | --- | --- | --- |
|  | *p*_Binomia_*_l_* | *z*_Full_ | *p*_Full_ | *z_Ha_*_lf_ | *p*_Half_ | Present | Absent | $\hat{d}$ |
| Right-Skewness Test | 0.031 | -5.128 | **<0.001** | -4.237 | **<0.001** | Yes | No | 0.161 |
| Flatness Test | >0.999 | 3.213 | >0.999 | 4.265 | >0.999 | Yes | No | 0.161 |
| Note: $\hat{d}$: p-curve’s estimate of true effect size | | | | | | | | |
| Binomial test: divides p-values into bins (>.025, <.025) | | | | | | | | |
| Half-Curve: considering the half range of p values (0 to .025) Full-Curve: considering the entire range of p-values (0 to .05) | | | | | | | | |

| Appendix Table 1. Search Strategy | | | | | |
| --- | --- | --- | --- | --- | --- |
| # | Database | Search String | Filters Applied | Searched Date (DD/MM/YYYY) | No. results |
| 1 | PubMed | ("Anxiety"[Mesh] OR "Anxiety" OR "anxiety disorder*" OR "social anxiety" OR "generalized anxiety disorder" OR "GAD" OR "Depression"[Mesh] OR "Depressive Disorder" [Mesh] OR "depression" OR "depress* disorder*" OR "MDD" OR "major depress* disorder*" OR "loneliness" OR "aloneness" OR "isolation" OR "social withdrawal" OR "social isolation" OR "stress" OR "Stress, Psychological"[Mesh] OR "mental stress" OR "psychological stress") AND ("epigenetic age" OR "epigenetic aging" OR "epigenetic clock" OR "biological clock" OR "biological age" OR "biological aging" OR "dna methylation age" OR "horvath" OR "hannum" OR "pheno"OR "grim" OR "dunedin") AND ((english[Filter]) AND (middleaged[Filter] OR aged[Filter] OR 80andover[Filter]) AND (2013:2024[pdat])) | Article Language: English; Age: Middle Aged: 45-64 years, Aged:65+ years, 80 and over: 80+ years  Year: 2013-2024 | 20/06/2025 | 628 |
| 2 | Scopus | ( TITLE-ABS-KEY ( "Anxiety" OR "anxiety disorder*" OR "social anxiety" OR "generalized anxiety disorder" OR "GAD" OR "depression" OR "depress* disorder*" OR "MDD" OR "major depress* disorder*" OR "loneliness" OR "Aloneness" OR "isolation" OR "social withdrawal" OR "social isolation" OR "stress" OR "psycholog* stress" OR "mental stress" ) AND TITLE-ABS-KEY ( "epigenetic clock" OR "epigenetic age" OR "epigenetic aging" OR "biological clock" OR "biological age" OR "biological aging" OR "dna methylation age" OR "horvath" OR "hannum" OR "pheno" OR "grim" OR "dunedin" ) ) AND PUBYEAR > 2012 AND PUBYEAR < 2025 AND ( LIMIT-TO ( LANGUAGE , "English" ) ) AND ( LIMIT-TO ( EXACTKEYWORD , "Aged, 80 And Over" ) OR LIMIT-TO ( EXACTKEYWORD , "Aged" ) OR LIMIT-TO ( EXACTKEYWORD , "Very Elderly" ) OR LIMIT-TO ( EXACTKEYWORD , "Middle Aged" ) OR LIMIT-TO ( EXACTKEYWORD , "Aging" ) OR LIMIT-TO ( EXACTKEYWORD , "Age" ) ) AND ( EXCLUDE ( DOCTYPE , "re" ) OR EXCLUDE ( DOCTYPE , "cp" ) | Language: English  Keyword: Aging, Aged, 80 And Over, Aged, Very Elderly, Middle Aged, Age  Document type: Excluded Review, Conference paper | 20/06/2025 | 778 |
| 3 | Embase | (**'anxiety'**:ab,ti OR **'anxiety disorder*'**:ab,ti OR **'social anxiety'**:ab,ti OR **'generalized anxiety disorder'**:ab,ti OR **'gad'**:ab,ti OR **'depression'**:ab,ti OR **'depress* disorder*'**:ab,ti OR **'mdd'**:ab,ti OR **'major depress* disorder*'**:ab,ti OR **'loneliness'**:ab,ti OR **'aloneness'**:ab,ti OR **'isolation'**:ab,ti OR **'social withdrawal'**:ab,ti OR **'social isolation'**:ab,ti OR **'stress'**:ab,ti OR **'psycholog* stress'**:ab,ti OR **'mental stress'**:ab,ti) AND (**'epigenetic clock'**:ab,ti OR **'epigenetic age'**:ab,ti OR **'epigenetic aging'**:ab,ti OR **'biological clock'**:ab,ti OR **'biological age'**:ab,ti OR **'biological aging'**:ab,ti OR **'dna methylation age'**:ab,ti OR **'horvath'**:ab,ti OR **'hannum'**:ab,ti OR **'pheno'**:ab,ti OR **'grim'**:ab,ti OR **'dunedin'**:ab,ti) AND [english]/lim AND ([middle aged]/lim OR [aged]/lim OR [very elderly]/lim) AND [2013-2025]/py | Language: English  Age: Middle aged, aged, elderly  Year:2013-2025 | 20/06/2025 | 415 |
| 4 | Web of Science (Wos) | (TS=("Anxiety" OR "anxiety disorder*" OR "social anxiety" OR "generalized anxiety disorder" OR "GAD" OR "depression" OR "depress* disorder*" OR "MDD" OR "major depress* disorder*" OR "loneliness" OR "Aloneness" OR "isolation" OR "social withdrawal" OR "social isolation" OR "stress” OR “mental stress” OR “psychological stress” )) AND TS=("epigenetic clock*" OR "epigenetic age" OR "epigenetic aging"OR "biological clock" OR "biological age" OR "biological aging" OR "dna methylation age" OR "horvath" OR "hannum" OR "pheno"OR "grim" OR "dunedin") AND PY=(2013 OR 2014 OR 2015 OR 2016 OR 2017 OR 2018 OR 2019 OR 2020 OR 2021 OR 2022 OR 2023 OR 2024 OR 2025) NOT DT=(Review) AND LA=(English) | Year:2013-2024  Language: English  Exclude-Document Types:Review Article | 20/06/2025 | 1477 |
| 5 | PsycInfo | (("Anxiety" or "anxiety disorder*" or "social anxiety" or "generalized anxiety disorder" or "GAD" or "depression" or "depress* disorder*" or "MDD" or "major depress* disorder*" or "loneliness" or "Aloneness" or "isolation" or "social withdrawal" or "social isolation" or "stress" or "mental stress" or "psychological stress") and ("epigenetic age" or "epigenetic aging" or "epigenetic clock" or "biological clock" or "biological age" or "biological aging" or "dna methylation age" or "horvath" or "hannum" or "phenoOR grim" or "dunedin")).mp. [mp=title, abstract, heading word, table of contents, key concepts, original title, tests & measures, mesh word] | Year:2013-26/07/2024  Language:  English  Age Groups:  360 Middle Age <age 40 to 64 yrs>  380 Aged <age 65 yrs and older>  390 Very Old <age 85 yrs and older> | 20/06/2025 | 171 |
| Note: The final search strategy focused on established epigenetic aging constructs and specific clock terminology (e.g., epigenetic age, epigenetic clock, Horvath, Hannum, PhenoAge, GrimAge). During search development, broader methylation-related terms (e.g., “DNA methylation”) were included but primarily retrieved studies addressing methylation levels rather than age-acceleration metrics and did not identify additional eligible records. Reference lists of included studies and relevant reviews were screened to minimize the risk of missed studies. | | | | | |

| Appendix Table 2. Table of Characteristics of Excluded Studies | | | |
| --- | --- | --- | --- |
| Study ID (Author, Year) | **Reasons of Exclusion** | **Study ID (Author, Year)** | **Reasons of Exclusion** |
| Aluzaite et al. 2021 | The study did not have any epigenetic age-related marker. | Lai et al.2025 | Abstract only |
| Andrasfay et al. 2023 | Lack quantification of mental risk | Vetter et al. 2022 | Two items from CES-d were used to quantify weakness, not depression |
| Andrasfay et al. 2023 | Stress was not quantified at present. | Martin et al. 2021 | While both Neighborhood environment and social cohesion were emphasized, there was not a psychological factor associated. |
| Apsley et al. 2023 | The study did not leverage the psychological factor of interest. | Morison et al. 2019 | The study did not have related psychological factor. |
| Berg et al. 2021 | This study did not meet the age criteria. | Mutambudzi et al. 2024 | The study only quantified the frequency of discrimination, without a quantification of stress. |
| Berg et al. 2022 | Both measurement and outcomes were not the interest of this study. | Noguchi et al. 2024 | This study did not meet the age criteria. |
| Berg et al. 2024 | The outcomes (e.g., incarceration exposure risk on depression) were not the scope of this review. | O'Shea et al. 2023 | The study touched upon the cognition-related outcomes, without LADS. |
| Bey et al. 2024 | The outcomes were heart failure. | Opsasnick et al. 2025 | Not current measurement |
| Beydoun et al. 2019 | Regression path reversed | Perez et al. 2021 | This study did not meet the age criteria. |
| Beydoun et al. 2022 | Exposure is discrimination, not psychological factors | Perez et al. 2022 | This study did not meet the age criteria. |
| Beydoun et al. 2024 | Outcome not related | Protsenko et al. 2021 | This study did not meet the age criteria. |
| Boks et al. 2015 | Neither age nor the outcome met the inclusion criteria. | Rentscher et al. 2023 | The study did not have any related psychological factors. |
| Bourassa et al. 2024 | Only PTSD was discussed, without a direct quantification of mental risk | Stephan et al. 2021 | No related psychological factors |
| Cha et al. 2025 | Abstract only | Sanford et al. 2025 | Pre-print only |
| Clausing et al. 2025 | Abstract only | Shindo et al. 2023 | This study did not meet the age criteria. |
| Das et al. 2022 | Social isolation, not loneliness | Shokhirev et al. 2025 | No psychological factor measurement. Training sets were terminal disease cohort |
| Davis et al.2025 | Abstract only | Vyas et al. 2019 | Included participants have bipolar disorders. |
| Das et al. 2023 | No related stress quantification | Vyas et al. 2021 | No related psychological factors |
| Farina et al. 2024 | Outcome not related | Wang et al. 2022 | The study did not have a quantification of stress, and only PTSD was discussed |
| Freni-Sterrantio., 2022 | Age range not met | Wang et al. 2023 | The study is a pre-print only |
| Gao et al. 2023 | Only the reversed relationship was reported | Wang et al. 2025 | Pre-print only |
| Guo et al. 2022 | A study looking at GAD whereas did not meet the age range criteria | Wang et al. 2024 | Not DNA m-based clock |
| Harvanek et al. 2021 | The study did not meet the age criteria. | Wolf et al. 2016 | The study emphasized PTSD, without a direct stress quantification |
| Hillmann et al. 2023 | Though the study underscored the social relationship, there was not any scaled psychological factor. | Wolf et al. 2018 | Only PTSD was discussed, without a direct quantification of mental risk |
| Jackson et al. 2023 | Outcome not related | Wolf et al. 2018 | PTSD only |
| Jung et al. 2023 | The age range criteria was not met. | Xu et al. 2024 | Pre-print only |
| Klopack et al. 2022 | No direct measurement of depression | Xu et al. 2024 | Pre-print only |
| Katrinli et al. 2023 | Only PTSD was discussed, without a direct quantification of mental risk | Yang et al. 2021 | Only PTSD was discussed, without a direct quantification of mental risk |
| Liang et al. 2022 | There was not any psychological related factor. | Zhang et al. 2024 | Psychological resilience is not an exposure of interest |
| Lynch et al. 2023 | Main outcome interest is cognition | Liang et al. 2022 | There was not any psychological related factor. |
| Lei et al. 2019 | The study did not have any related psychological factors. | Li et al. 2025 | No related regression path |

| Appendix Table 3. Study-Level Classification of Associations Between Psychological Exposures and Epigenetic Clocks | | | | |
| --- | --- | --- | --- | --- |
| *Psychological domain* | *Clock Type* | *Positive Significant (Study IDs)* | *Negative Significant (Study IDs)* | *Non-Significant (Study IDs)* |
| Stress | GrimAge | Bergquist et al. (2022); Skinner et al. (2023); Suglia et al. (2024) |  | Freni-Sterrantio et al. (2022); Nwanaji et al. (2023); Vetter et al. (2022) |
| Stress | PhenoAge | Boen et al. (2023) | Vetter et al. (2022) | Freni-Sterrantio et al. (2022); Nwanaji et al. (2023) |
| Stress | HorvathAge | Zannas et al. (2015) | Nwanaji et al. (2023) | Freni-Sterrantio et al. (2022); Markon et al. (2024); Vetter et al. (2022) |
| Stress | HannumAge |  | Nwanaji et al. (2023) | Freni-Sterrantio et al. (2022); Markon et al. (2024); Vetter et al. (2022) |
| Stress | DunedinPACE / PoAm | Markon et al. (2024); Suglia et al. (2024) |  |  |
| Stress | IEAA |  | Nwanaji et al. (2023) |  |
| Stress | EEAA | Skinner et al. (2023) | Nwanaji et al. (2023) |  |
| Stress | SkinBloodAge | Suglia et al. (2024) | Nwanaji et al. (2023) |  |
| Stress | KDM Biological Age | Boen et al. (2023) |  |  |
| Depression | GrimAge | Joshi et al. (2024); Liu et al. (2022); Protsenko et al. (2021); Wang et al. (2024) |  | Robert et al. (2024); Tanifuji et al. (2023) |
| Depression | PhenoAge | Liu et al. (2022); Wang et al. (2024) |  | Joshi et al. (2024); Robert et al. (2024); Tanifuji et al. (2023) |
| Depression | HorvathAge |  |  | Joshi et al. (2024); Liu et al. (2022); Robert et al. (2024); Tanifuji et al. (2023); Wang et al. (2024) |
| Depression | HannumAge | Liu et al. (2022); Wang et al. (2024) |  | Joshi et al. (2024); Robert et al. (2024) |
| Depression | DunedinPACE / PoAm | Sorlí et al. (2024) |  | Robert et al. (2024) |
| Depression | LevinAge | Brown et al. (2017) |  |  |
| Depression | Custom MBD-Seq Clock | Han et al. (2018) |  |  |
| Depression | MOPA | Wang et al. (2024) |  |  |
| Depression | SkinBloodAge |  |  | Tanifuji et al. (2023) |
| Depression | Cortical BrainAge |  |  | Simons et al. (2023) |
| Depression | IEAA |  |  | Liu et al. (2022) |
| Depression | EEAA | Liu et al. (2022) |  |  |
| Loneliness | GrimAge | Freilich et al. (2024) [UCLA]; Freilich et al. (2024) [3-item] |  |  |
| Loneliness | DunedinPACE / PoAm | Beach et al. (2022); Freilich et al. (2024) [3-item] |  |  |
| Loneliness | EAA Average | Freilich et al. (2024) [UCLA]; Freilich et al. (2024) [3-item] |  |  |
| Loneliness | HorvathAge |  |  | Freilich et al. (2024) |
| Loneliness | HannumAge |  |  | Freilich et al. (2024) |
| Loneliness | PhenoAge |  |  | Freilich et al. (2024) |
| Loneliness | Cortical BrainAge | Simons et al. (2023) |  |  |
| Note: Counts reflect study-level classification per clock using the adjusted model when available. Each study–clock combination was categorized as positive significant (p < 0.05), negative significant (p < 0.05), or non-significant. | | | | |

## **Appendix A.** NEWCASTLE-OTTAWA SCALE (NOS)

**(Adapted** for Observational study)

Author Note: Included studies either used data from an existed cohort or recruited participants on their own. I choose not to include ‘sample size’ item as they do not have control over this sample size. *Outcome of interest*, EAA, is a floating/continuous variable. This form is tailored to align with such circumstance.

**Selection (Maximum 4 starts)**

1) **To what extent do include participants representative of the general population (1 star)**

a) Truly representative of the general mid-aged and older population (e.g., random sampling from the general population) *

b) Somehow representative of the target population (e.g., sample drawn from a specific region or demographic group) *

c) Participants were selectively recruited from hospital/clinic

d) Sampling strategy or demographic is missing

2) **Measurement of Exposure: Psychological Adversities Ascertainment of exposure (1 star)**

a) Respective mental burden was assessed with validated measurement tools with item presented if adapted form has been used. *

b) Measurement tools are not validated or lack sufficient detail

**3) Was the source of participants clearly described?**

a) The study clearly stated source of the recruited participants (e.g., community-based cohort, national based cohort, clinical registry, etc.) *

b) No (source unclear not stated)
4) **Measurement of Outcome: Epigenetic Aging (1 star)**

a) Multiple validated epigenetic clocks (> 2) are employed. Details of selected CpGs and algorithm are presented, if self-proposed epigenetic clock has been used, along with description of laboratory methods. *

b) Only one or non-validated epigenetic clock is used, or details of selected CpGs are missing when self-proposed epigenetic clock has been used

**Comparability: (Maximum 2 stars)**

1) **Appropriate statistical analysis was conducted to examine the effects of potential confounders (0-2 stars)**

a) Study controls for the most important cofounder (e.g., chronological age, sex) and additional relevant factors (e.g., lifestyle) **

b) Study controls for the most important confounders (e.g., chronological age, sex) only. *

c) No adjustment for confounders or confounders is inadequately addressed.

**Outcome: (Maximum 3 stars)**

1) **Assessment of the outcome (1 star)**

a) Detailed protocol of bio-sample collection (e.g., venous blood, saliva) and methodology of quantifying the epigenetic age are provided 1 score *

b) Methodology is incomplete or not described

2) **Statistical Analysis Transparency (1 star)**

a) Employed statistical tests are appropriate, clearly described, and results are presented transparently, including model selection, covariates, statistical power. *

b) Statistical test is incomplete, not appropriate or not described.

3) **Was follow-up long enough for changes to occur?**

a) Yes, if at least a 5-year gap for study with n = 2 timepoints; at least a 2-year gap for study with n > 2 timepoints) *
b) No

| Appendix B. Summary of Study Quality assessment | | | | | | | | | |
| --- | --- | --- | --- | --- | --- | --- | --- | --- | --- |
| Study ID (Author, Year) | **Selection** | | | | **Comparability** | **Outcome** | | | **Total** |
|  | *Sample Representativeness* | *Assessment of Exposure* | *Source of participants* | Outcome measurement | *Confounders adjustment* | *Assessment of outcome* | *Statistical Analysis Transparency* | *Follow-up adequacy* |  |
| Beach et al. (2022) | ★ | ★ | ★ |  | ★★ | ★ | ★ | ★ | ★★★★★★★★ |
| Bergquist et al. (2022) |  | ★ | ★ |  | ★★ | ★ | ★ |  | ★★★★★★ |
| Boen et al. (2023) | ★ |  | ★ | ★ | ★★ | ★ | ★ |  | ★★★★★★★ |
| Nwanaji et al. (2023) | ★ | ★ | ★ | ★ | ★★ | ★ | ★ |  | ★★★★★★★★ |
| Freni et al. (2022) | ★ | ★ | ★ | ★ | ★★ |  | ★ |  | ★★★★★★★ |
| Markon et al. (2024) |  | ★ | ★ | ★ | ★★ | ★ | ★ |  | ★★★★★★★ |
| Skinner et al. (2023) | ★ | ★ | ★ | ★ | ★★ | ★ | ★ |  | ★★★★★★★★ |
| Suglia et al. (2024) |  | ★ | ★ | ★ | ★ | ★ | ★ |  | ★★★★★★ |
| Vetter et al. (2022) | ★ | ★ | ★ | ★ | ★★ |  | ★ |  | ★★★★★★★ |
| Zannas et al. (2015) | ★ | ★ | ★ |  | ★★ | ★ | ★ |  | ★★★★★★★ |
| Han et al. (2018) | ★ | ★ | ★ | ★ | ★★ | ★ | ★ |  | ★★★★★★★★ |
| Joshi et al. (2024) | ★ | ★ | ★ | ★ | ★★ | ★ | ★ |  | ★★★★★★★★ |
| Liu et al. (2022) | ★ | ★ | ★ | ★ | ★★ | ★ | ★ | ★ | ★★★★★★★★★ |
| Patrick et al. (2017) | ★ | ★ | ★ |  |  | ★ | ★ |  | ★★★★★ |
| Protsenko et al. (2021) | ★ | ★ | ★ |  | ★ | ★ | ★ |  | ★★★★★★ |
| Roberts et al. (2024) | ★ | ★ | ★ | ★ | ★★ |  | ★ | ★ | ★★★★★★★★ |
| Simons et al. (2023) | ★ | ★ | ★ | ★ | ★ | ★ | ★ |  | ★★★★★★★ |
| Tanifuji et al. (2023) |  |  | ★ | ★ | ★ | ★ |  |  | ★★★★ |
| Wang et al. (2024) | ★ | ★ | ★ |  | ★★ | ★ | ★ |  | ★★★★★★★ |
| Sorlí et al. (2024) | ★ | ★ | ★ |  | ★★ | ★ |  |  | ★★★★★★ |
| Freilich et al. (2024) | ★ |  | ★ | ★ | ★★ | ★ | ★ |  | ★★★★★★★ |
| Freilich et al. (2024) | ★ | ★ | ★ | ★ | ★★ | ★ | ★ |  | ★★★★★★★★ |
| *Note:* Newcastle-Ottawa Scale adapted for observational studies  Quality was assessed based on three sections: Selection; Comparability; Outcome.  ^a^ Each column within the section has a maximum of one star obtainable, except for comparability  ^b^ A maximum of two stars can be awarded to Comparability  Score cutoff: ≥ 7 (high quality); < 7 but ≥ 5 (moderate quality); < 5 (low quality) | | | | | | | | | |
